# Non-steroidal anti-inflammatory drugs and biomarkers: A new paradigm in colorectal cancer

**DOI:** 10.3389/fmed.2023.1130710

**Published:** 2023-03-06

**Authors:** Gowhar Rashid, Nihad Ashraf Khan, Deena Elsori, Andleeb Rehman, Tanzeelah, Haleema Ahmad, Humaira Maryam, Amaan Rais, Mohd Salik Usmani, Asaad Ma Babker, Mohammad Azhar Kamal, Wael Hafez

**Affiliations:** ^1^Department of Amity Medical School, Amity University, Gurugram, India; ^2^Department of Biosciences, Jamia Millia Islamia, Central University, New Delhi, India; ^3^Faculty of Resillience, Deans Office Rabdan Academy, Abu Dhabi, United Arab Emirates; ^4^Department of Biotechnology, Shri Mata Vaishno Devi University, Katra, India; ^5^Department of Biochemistry, University of Kashmir, Srinagar, India; ^6^Department of Biochemistry, Faculty of Life Sciences, AMU, Aligarh, India; ^7^The Department of Surgery, Faculty of Medicine, JNMCH, AMU, Uttar Pradesh, India; ^8^Department of Medical Laboratory Sciences, Gulf Medical University, Ajman, United Arab Emirates; ^9^Department of Pharmaceutics, College of Pharmacy, Prince Sattam Bin Abdulaziz University, Alkharj, Saudi Arabia; ^10^Department of Internal Medicine, NMC Royal Hospital, Abu Dhabi, United Arab Emirates; ^11^The Medical Research Division, Department of Internal Medicine, The National Research Center, Ad Doqi, Egypt

**Keywords:** colorectal cancer, biomarkers, NSAIDs, colonoscopy, KRAS, chemoprevention, COX-pathways, statins

## Abstract

Colorectal cancer is a sporadic, hereditary, or familial based disease in its origin, caused due to diverse set of mutations in large intestinal epithelial cells. Colorectal cancer (CRC) is a common and deadly disease that accounts for the 4^th^ worldwide highly variable malignancy. For the early detection of CRC, the most common predictive biomarker found endogenously are KRAS and ctDNA/cfDNA along with *SEPT9* methylated DNA. Early detection and screening for CRC are necessary and multiple methods can be employed to screen and perform early diagnosis of CRC. Colonoscopy, an invasive method is most prevalent for diagnosing CRC or confirming the positive result as compared to other screening methods whereas several non-invasive techniques such as molecular analysis of breath, urine, blood, and stool can also be performed for early detection. Interestingly, widely used medicines known as non-steroidal anti-inflammatory drugs (NSAIDs) to reduce pain and inflammation have reported chemopreventive impact on gastrointestinal malignancies, especially CRC in several epidemiological and preclinical types of research. NSAID acts by inhibiting two cyclooxygenase enzymes, thereby preventing the synthesis of prostaglandins (PGs) and causing NSAID-induced apoptosis and growth inhibition in CRC cells. This review paper majorly focuses on the diversity of natural and synthetic biomarkers and various techniques for the early detection of CRC. An approach toward current advancement in CRC detection techniques and the role of NSAIDs in CRC chemoprevention has been explored systematically. Several prominent governing mechanisms of the anti-cancer effects of NSAIDs and their synergistic effect with statins for an effective chemopreventive measure have also been discussed in this review paper.

## 1. Introduction

Colorectal cancer remains one of the fourth most common malignancies worldwide after lung, liver, and stomach cancer. It majorly develops after the age of 50 whereas, a dramatic increase in the younger generation has been observed with an expected increase rate of 140% by the year 2030. A significant disparity in the incidence and survival rates of CRC between developed and developing countries depicts a difference in socioeconomic development (1).Genetic inheritance has been proved to play an important role in the development of CRC, with men being the major targets. Apart from genetic predisposition, lifestyle factors such as inactivity, type-2 diabetes mellitus (TDM), alcohol consumption, smoking, and obesity also influence the risk of CRC (2). Familial adenomatous polyposis and lynch syndrome are the two most prominent inherited syndromes that account for approximately 5% of all CRC. The accumulation of genetic mutations results in the transformation of normal colonic epithelium to a precancerous lesion and ultimately to invasive carcinoma over 10-15 years. Whereas people having adenomatous polyps or polyps with villous or tubulovillous dysplasia are at higher risk of developing synchronous and metachronous CRC primary cancer. Unfortunately, people who survived cancer at a childhood age and received abdominal radiation are at higher risk of developing CRC thus, it is recommended to adopt a screening session after 10 years or at the age of 35 (3). Hence, the early detection and removal of preformed or developing polyps will eliminate the chances of CRC. Polyps which are hamartomatous and serrated have also proven to be responsible for leading to CRC. The molecular pathways such as chromosomal instability, mismatch repair and hypermethylation has been attributed to the major pathways linked to CRC (4). Adenocarcinomas accounts for more than 90% of CRC whereas adenosquamous, spindle, squamous and undifferentiated are frequently not seen. Among the treatments for CRC, surgical resection is commonly adopted for localized non-metastatic stage CRC. Additionally, palliative systemic chemotherapy and the use of NSAIDs as chemoprevention are offered to non-surgical candidates and may prove to be a curative option (5).

Surgical removal of polyps and increasing death of CRC requires the demand of risk assessment, screening, differential diagnosis, prognosis determination, treatment response prediction, and disease progression monitoring. These potentialities are determined with the help of biomarkers in oncology. Biomarkers help in biological observation, which ideally predicts the endpoint or intermediate outcome of a disease at an early stage where it is difficult to be observed (6). Biomarkers must undergo a thorough evaluation, including analytical validation, clinical validation, and assessment of clinical utility, before being incorporated into routine clinical care because of the crucial role they play at all stages of the disease (7). In CRC treatment biomarkers, molecular pattern act as a tool for the early detection of colorectal cancer. These biomarkers play an important role in the early detection and well-individualized treatment of people suffering from cancer. The various categories of biomarkers are predictive, prognosis, and diagnostic which help to determine the progression and recurrence of cancer whereas, it also proves to be an effective therapeutic target (8). The detail view of various biomarkers along with their potentiality in CRC has been mentioned systematically in the next section.

After an early detection of CRC, the intervention of therapeutics to curb the progression of colorectal cancer becomes an important task. Hence, NSAIDs are believed to have a chemopreventive impact on gastrointestinal malignancies, and more especially, on colorectal cancer, according to a significant body of data from epidemiological and preclinical research (9). Non-steroidal anti-inflammatory drugs (NSAIDs) are a group of chemical compounds that are typically unrelated yet have some therapeutic qualities and side effects. They are among the most widely used medicines in the world and have potent analgesic, antipyretic, and anti-inflammatory properties (10). NSAIDs are among the most widely used medications, supporting their inclusion on the WHO’s Model List of Essential Medicines due to their effectiveness in lowering pain and inflammation (11). They primarily work by inhibiting two cyclooxygenase enzymes, which stop the production of prostaglandins (PGs). Numerous cellular activities, including gastrointestinal cytoprotection, hemostasis and thrombosis, inflammation, renal hemodynamics, cartilage turnover, and angiogenesis, depend heavily on PGs. A lot of different illnesses’ pathophysiologies are heavily influenced by inflammation. PGs, coagulation cascade-derived peptides, interleukin IL-2, IL-6, and tumor necrosis factor (TNF) are among the inflammatory mediators whose production and activity are affected by NSAIDs (12). Long-term use of NSAIDs has also been linked to renal illness, which can cause both acute and chronic abnormalities in kidney function (13). The US Food and Drug Administration (FDA) was led by these consequences to issue a scientific statement in 2005 that emphasized, “the necessity of utilizing the lowest effective dose for the shortest time feasible if therapy with an NSAID is necessary for an individual patient” (14).

## 2. Biomarkers

According to the National Cancer Institute, a biomarker is a biological molecule found in blood, other body fluids, or tissues that is a sign of a normal or abnormal activity, as well as of a condition or disease, such as cancer (NCI). A patient with the condition can frequently be distinguished from a healthy person using biomarkers. The adjustments could be brought on by post-translational modifications, somatic or germline mutations, transcriptional changes, or other factors. Proteins (such as an enzyme or receptor), nucleic acids (such as a microRNA or other non-coding RNA), antibodies, and peptides are only a few examples of the wide variety of biomarkers. A few examples of the kinds of alterations that can be regarded biomarkers are changes in gene expression, proteomic signatures, and metabolomic signatures. In order to be analyzed non-invasively and serially, biomarkers can be detected in the circulation (whole blood, serum, or plasma), excretions or secretions (stool, urine, sputum, or nipple discharge), or they may be formed from tissues, necessitating a biopsy or specialized imaging. Sequence variations in germ-line DNA recovered from whole blood, sputum, or buccal cells are examples of inherited genetic biomarkers. Mutations in DNA extracted from tumor tissue are examples of somatic genetic biomarkers (8). Briefly tabulated certain biomarkers and their significance in various type of cancer in Table 1. Prostate-specific antigen (PSA) is a frequently employed but contentious biomarker for screening (22). Biomarkers can be used to assess a patient’s prognosis, or the likelihood of the disease returning without regard to treatment, after a cancer diagnosis. More lately, the prognosis for specific malignancies is being determined using modern methods. Additionally, biomarkers can be used to modify the response to a particular therapy, or as “predictive factors,” or to determine which therapy is most likely to be successful. Because somatic mutations in KRAS are linked to poor response to anti-epidermal growth factor receptor (EGFR) focused therapy, KRAS is a predictive biomarker for colorectal cancer (23).

**Table 1:** Biomarkers and their significance in various type of cancers.

S. no.	Type of cancer	Biomarkers	Significance	Drawbacks	References
1.	Lung cancer	- Plasma CD4 levels	- Identification of benign lung nodules-89% Specificity	- No validation for high-risk individuals-Mild CT screening trial	(15)
		- miRNA	- 81 % specificity - 87% sensitivity	-	
		- ctDNA - CTCs	- Tumor shed Product	- Advanced tumor stages - Sensitivity 57%	
		- Blood antigens: CYFRA 21-1, CEA, NSE, SCC-Ag	- 88–95% specificity	- Multi-antigen approach is required	
2.	Liver cancer	- GP73 - CA19-9 - GPC3 - Hep Par 1 - Gs - Arg 1	- Helps in Diagnosis - Prominent Indicators - Average 95–100% specificity	- Combined identification is required	(16)
3.	Stomach cancer	- CEA - CA19-9 - CA72-4 - CA125 - HER2	- Helps in early detection - Involved in diagnosis and prognosis	- HER2-Prognosis is not established	(17)
4.	Colorectal cancer	- KRAS - BRAF	- 94–98% specificity - Prognostic and predictive factor	-	(18)
		- PTEN	-Predictive factor		
		- TP53	- 58% sensitivity - 88% specificity		
		- CEA	- Screening - Prognostic factor		
5.	Ovarian cancer	- CA125	- Predicts prognosis EOC	- Low sensitivity 67.39% - No clinical value	(19)
		- HE4	- Detection of Endometrioid - 91.4% specificity	-	
		- OPN	- Early detection	-	
6.	Prostate cancer	- PCA3	- Significant biomarker - Approved biomarker - Specificity 88%	-	(20)
		- PSA glycoforms - MPRSS2-ERG	- Detection, potential new biomarkers	- Not approved yet	
7.	Breast cancer	- BRCA 1/2	- 98-100% specificity - Early diagnostic and prognosis of cancer	-	(21)

Overexpression of the estrogen receptor in breast cancer predicts sensitivity to anti-endocrine therapy like tamoxifen (24) whereas overexpression of the HER2 gene or gene amplification in gastric and breast cancers predicts response to anti-Her2 drugs like trastuzumab. Chemotherapy sensitivity and resistance assays, which have been researched in a variety of tumor types, are potential somatic biomarkers for predicting response to therapy. These assays are offered commercially and have been the subject of numerous published clinical investigations (25).

Biomarkers can be utilized to identify early disease recurrence in patients who have finished adjuvant therapy before they experience symptoms. For instance, serial monitoring of CEA after adjuvant treatment for colon cancer is done to look for liver metastases while they are still treatable and resectable (26). Similar to this, beta HCG, lactate dehydrogenase, and alpha-fetoprotein are serially examined in non-seminomatous germ cell tumors to look for early disease recurrence. Additionally, biomarkers can be used to monitor the efficacy of treatment in the context of metastatic disease. Circulating soluble protein tumor indicators such CEA, PSA, CA125, the MUC1 antigens CA15, CA27.29, and CA19, as well as the efficiency of palliative care in metastatic colorectal, prostate, ovarian, breast, and pancreatic cancers, are suggested (27).

### 2.1. Synthetic biomarkers

**Figure 1:**
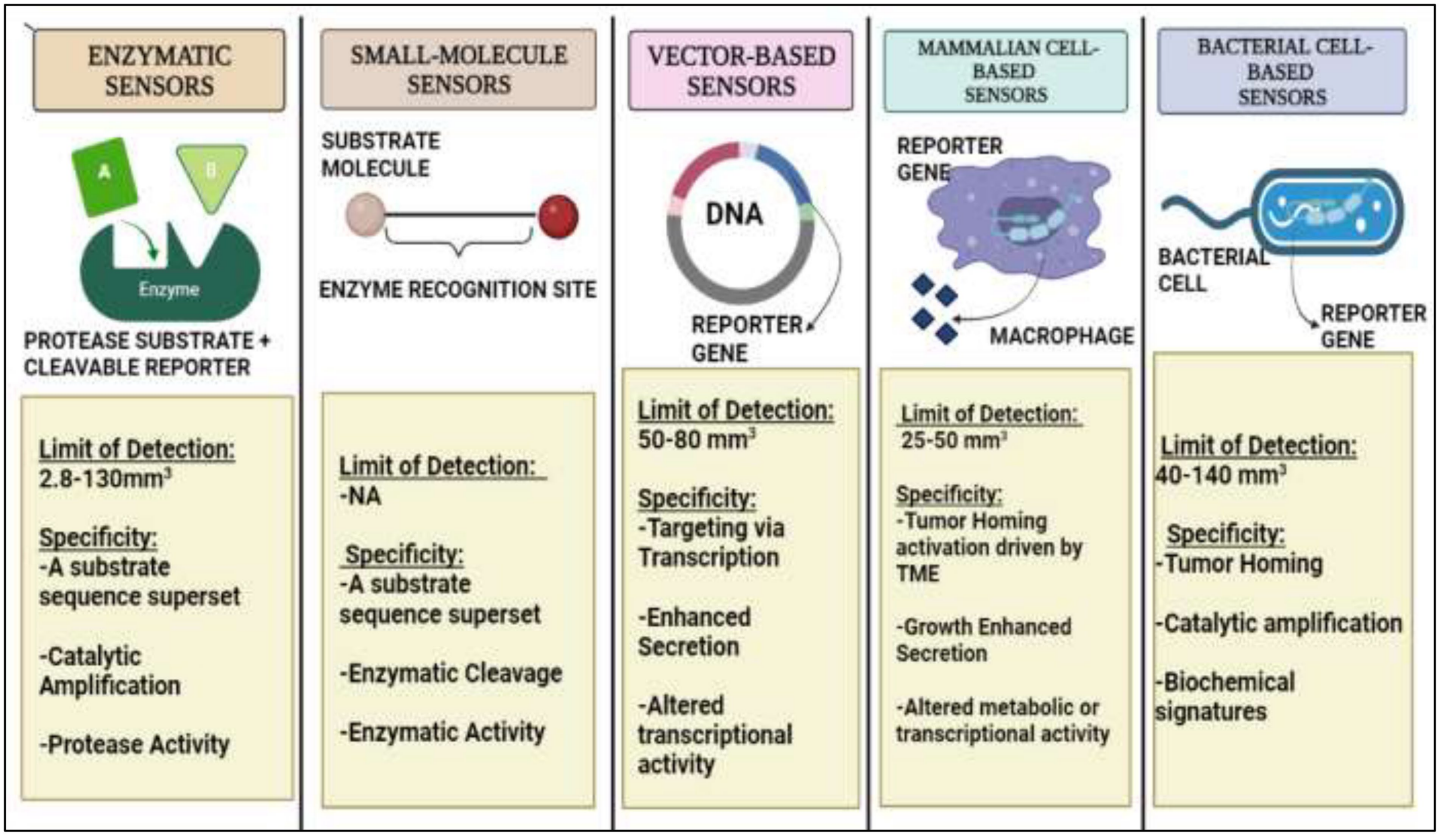
A detailed diagrammatic overview of Synthetic Biomarkers on the basis of their activity and various methodologies for endogenous administration.

Some researchers are adopting a different strategy rather than depending on endogenous signals, which come from the body. They are tricking a tumor into secreting synthetic biomarkers that can be detected in biofluids while the tumor is still undetectable by any existing technique by using cunning engineering technologies and tumor-specific biological knowledge. When ingested, the exogenously supplied bioengineered sensors can send out a signal indicating the presence of cancer cells. These techniques have successfully detected significantly lower tumor sizes in animal models (28). The biological, physiological, and statistical constraints of endogenous biomarkers serve as a justification for the development of synthetic biomarkers. Endogenous biomarkers such as proteins in the pool of blood and having varying secretion rates are difficult to detect due to short periods of retention and frequent clearance from circulation (29). These represent a new class of diagnostics that use bioengineered sensors such as molecular probes or genetically encoded vectors that take the advantage of the potentially dysregulated characteristics of early stage tumors or their precursors which could become lethal, inside the body to scan for early stage tumors and amplify illness signals to levels that may be greater than those of shed biomarkers detectable from body fluids such as blood and urine. Several imaging techniques also employ synthetic biomarker approach including reporter gene imaging, in which an exogenous molecular tracer (such as a positron emitting probe) is systematically infused (29). Synthetic biomarkers on the basis of their activities are called protease-activated synthetic biomarkers that are particularly effective molecular amplifiers. Apart from it vector-based, mammalian cell-based, and bacterial cell-based synthetic biomarkers are also employed on the basis of their advantages (Figure 1). Moreover, some preclinical studies have reported the potential use of activity-based sensor composed IONPs synthetic biomarkers for early detection of LS174T colorectal cancer (30).

## 3. Biomarkers for early detection of colorectal cancer

Colorectal cancer is treatable if caught early enough. As a result, early identification of colorectal cancer can minimize mortality. The categories of colorectal biomarkers that are now studied include proteins, mutated and methylated DNA, RNAs that are mostly microRNAs, volatile organic chemicals, alterations, and variations in gut microbiota makeup. It is generally known that early-onset CRC is becoming more common and is more deadly among those under the age of 50. These patterns have prompted thorough research aimed at clarifying the epidemiology and characteristics of early-onset CRC as well as formulating tactics for early identification and prevention. It is generally known that during the past 30 years, early-onset CRC incidence has grown globally (31). The identification of blood-based biomarkers may be a useful screening method for CRC due to how simple it is to donate or collect blood. A significant percentage of sporadic, non-hereditary malignancies have genetic abnormalities in the initial phases of carcinogenesis. Large numbers of these aberrant cells are shed from the expanding tumor, and their cell-free nucleic acids can be found in biological effluents, especially in urine, serum, and faeces. To promptly detect genetic disorders, molecular biomarkers with higher sensitivity and specificity than the faecal occult blood test (FOBT) or faecal immunochemical test (FIT) can be utilized (32). The biomarkers can be grouped into broader categories: Blood, Tissue, Stool, and Others.

### 3.1. Blood biomarkers

1. **Tumor cells in circulation**: According to a recent study, a limited fraction of circulating tumor cells (CTCs) with the ability to cause metastasis include those that express the molecules; EpCAM, CD44, CD47, and MET. It has been found that individual CTCs from the same patient have different KRAS, BRAF, and PIK3CA mutations. CTC are detected using flow cytometry and immunocytochemical analysis are also highly sensitive methods to detect biomarkers for CRC (33).
2. **Tumor DNA in circulation**: There is circulating tumor DNA or ctDNA called cell-free DNA (cfDNA) in cancer patients that are a diagnostic biomarker for CRC (34). cfDNA contains mutations, methylation, microsatellite instability, and loss of heterozygosity that contribute to tumor-specific alterations (35). There is a high concentration of cfDNA in neoplastic disease. The ctDNA/cfDNA is considered a novel biomarker for the early detection of colorectal malignancies.
3. **Micro RNAs in circulation**: Non-coding RNAs subclass contains more than 38,500 microRNAs in human beings discovered so far. Circulating upregulated or downregulated miRNAs like miR-18a, miR-31, miR-145, miRNA-486, miRNA-320, miRNA-451, etc., are sensitive biomarkers for CRC (36). Other Biomarkers like ctDNA, mSEPT9 DNA, miR-31, miR-141, miR-224-3p, miR-576-5p, miR-4669, miR-21, exosomal miR-548c-5p, lncRNA CRNDE-h, etc. were identified as biomarkers of CRC. Dysregulation of miRNAs is frequent in CRC and hence are potential biomarkers. RT-qPCR and next-generation sequencing (NGS) are a few methods to detect these miRNAs.
4. **DNA methylation-based biomarkers**: In CRC the most frequent process to occur as compared to genetic mutation is the methylation of the CpG island of the promoter. More than 600 hypermethylated genes have been identified so far. Among these, the best-known biomarker for CRC is the *SEPT9* methylated DNA. There are 13 genes in the *SEPT* gene family, which is located on chromosome 17q25 in the human genome (37).
5. **Long non-coding RNA-based biomarkers**: Long noncoding RNAs (lncRNAs) have been shown to be stable in blood and to have diagnostic potential during the past 10 years (38). Through processes, such as chromatin remodeling, chromatin interaction, competing endogenous RNAs, and natural antisense transcripts, they can affect cancer cells (39). Because lncRNAs may pass across cell membranes, they can be discovered in a variety of bodily fluids, including blood, plasma/serum, and urine. Various biomarkers used for CRC detection based on these lncRNAs are CCAT1, HOTAIR, LOC285194, RP11-462C24.1, BCAR4, BLACAT1, UCA1, 91H, PVT-1, MEG3, ATB, CCAT1, NEAT1, etc.
6. **Others**: Pyruvate kinase M2 (PKM2), an isoform of pyruvate kinase enzyme is reported as overexpressed in CRC. PKM2’s great sensitivity makes it appear like a viable blood and fecal biomarker for CRC screening (40).

### 3.2. Tissue biomarkers

1. **Transcription factors**: The caudal type homeobox transcription factor (CDX2) is one of many transcription factors that contribute to the development and differentiation of the intestine (41). It is a widely used immunomarker for CRC as it is a tumor suppressor gene in CRC and its expression is lacking in CRC cases. Another transcription factor special AT-rich sequence-binding protein 2 (SATB2) regulates skeletogenesis and is a CRC biomarker with a positivity rate of 83.7% of stage III/IV colorectal adenocarcinomas, 91.4% of stage II and 92.4% of stage I of this malignancy (42).
2. **Transmembrane glycoproteins**: The A33 antigen, a type I transmembrane glycoprotein of the immunoglobulin superfamily, is expressed in the basolateral membranes of both proliferating cells in the lower regions of the crypts and differentiating cells in the upper regions of the crypts, as well as in 95% of colon tumors in the colon and small intestine (10). Another glycoprotein, a member of the cadherin superfamily, cadherin-17 (CDH17) is a calcium-dependent transmembrane glycoprotein (43). In normal, metaplastic, and neoplastic tissues of the gastrointestinal tract, CDX2 binds to elements in the 50 flanking regions of the gene to regulate this cadherin’s transcription. With a specificity of 50–83.8% and a sensitivity of 96–100%, CDH17 is a helpful immunohistochemical marker for the identification of primary and metastatic colorectal adenocarcinomas (44).
3. **Telomerase**: Telomeres, which guard the ends of chromosomes, have certain hexameric repeats (TTAGGG)n in them. They control the longevity of cells and chromosomal integrity. A telomere-specific reverse transcriptase (hTERT) found in telomerase is similar in structure and function to viral transcriptase. The replicative capacity of CRCs and the risk of recurrence are increased by the overexpression of hTERT (45). hTERT appears to be a recurrent biomarker that may be utilized to track systemic treatment responses.
4. **Cytokeratins**: The intermediate filament-forming protein known as cytokeratins is found in the cytoplasmic cytoskeleton. The only cells that exhibit them are epithelial cells. Numerous cellular processes, including cell size determination, apical-basal polarization, protein translation regulation, organelle location, and membrane protein targeting, are regulated by cytokeratins (46). In CRC diagnoses, cytokeratins are frequently utilized as immunohistochemistry markers. Various cytokeratins involved in the prognosis expressed in CRC patients are cytokeratin 7, cytokeratin 20, cytokeratin 20+/cytokeratin 7-, cytokeratin 15, and cytokeratin 18 (47).

### 3.3. Stool biomarkers

As the exfoliating tumor cells first occur in the large intestine or rectal lumen during colorectal carcinogenesis, stool specimens are more suitable for the early identification of CRC than blood samples (48). The presence of stool biomarkers has resulted in the early detection of CRC. The guaiac-based fecal occult blood testing (gFOBT) and fecal immunochemical test are mostly used for the screening of rectal blood loss, which is the biomarkers in the stool. The fecal microRNA-106a test is used to detect mRNAs in stool (37). Tumor suppressor genes are rendered inactive by hypermethylation at every stage of carcinogenesis, from polyps to colorectal adenocarcinomas. Many genes, particularly those in the promoter region, are methylated in CRC, including APC, MLH1, MGMT, SFRP1, SFRP2, CDK2A, TIMP3, VIM, SEPT, CDH1, and HLTF (49). There are numerous methylated DNA stool biomarkers used in CRC like SFRP methylation, CDKN2A, MGMT methylation, Vimentin methylation, NDRG4 methylation, BMP3 methylation, K-*ras* mutation, hypermethylated SCNA, etc.

## 4. Techniques and current advancements in biomarkers for early detection of CRC

### 4.1. Techniques for early detection of CRC

Early detection and screening for CRC is necessary and could potentially be lifesaving in many cases, as the symptoms of CRC often tend to develop late in the natural course of the disease (50). Multiple methods can be used to screen and perform early diagnosis of CRC, each with its associated advantages and disadvantages. The most important feature of these tests is the test’s sensitivity, and to a certain degree, its specificity (51).

#### 4.1.1. Colonoscopy

Endoscopic procedures involve passing a camera attached to a long flexible tube into the gut of the patient. These procedures can be used to visualize and non-surgically remove adenomas and early cancers (52). Currently, colonoscopy is the most prevalent method for diagnosing CRC or confirming the positive result from other screening methods, with most doctors suggesting regular colonoscopy at a gap of 10 years for patients over the age of 45. A colonoscopy can be performed to spot and remove pre-cancerous lesions and tumors across the entire large bowel (53). Its sensitivity for CRC detection is around 95%, and for advanced adenomas (about 10 mm in diameter) its sensitivity is around 88–98%. It has been seen in case–control studies that with the use of colonoscopy there was a decline of about 53–72% in the incidence of CRC and a 31% decline in CRC-associated mortalities (54). But colonoscopy has its associated disadvantages, like high dependency on the operator, significant burden to the patient, expensive nature, post-colonoscopy CRC risk, etc. (55).

#### 4.1.2. Sigmoidoscopy

Flexible sigmoidoscopy (FSIG) enables the endoscopic examination of the rectum and, distal colon (56). FSIG is most performed without sedation, unlike colonoscopy (57). Concerning colonoscopy, has its advantages it requires less intestinal preparation, takes less time, causes less discomfort for the patient without anesthesia, has fewer complication rates, and is cheaper (58). The common risk associated includes bleeding and perforations (59). Within this test’s reach, the sensitivity and specificity for large adenomas and CRC have been found to be 95 and 87%, respectively.

#### 4.1.3. Colon capsule endoscopy

Colon capsule endoscopy or CCE is a recent development in the field of CRC screening and involves swallowing a wireless camera, which has the size of a pill, which moves along the GI tract taking images of its surroundings (60). For advanced neoplasia, 10 mm or larger, the CCE-2 has a sensitivity of 76.7% and a specificity of 90.7% (61). g-FOBT and fecal immunochemical tests Guaiac Faecal Occult Blood Tests or g-FOBT involve the testing of stool for the presence of blood in it. The stool sample is tested using peroxidase enzyme for the presence of the heme group using a guaiac card. A positive g-FOBT necessitates a follow-up colonoscopy test (12). Fecal Immunochemical Tests or FITs incorporate antibodies that specifically bind to the globin protein of hemoglobin. Thus, like g-FOBT, they also search for the presence of blood in the stool of the patient (62). The biggest advantage of such stool-based tests is the ease of use. An issue with these techniques is that most polyps do not bleed. Thus, their presence goes undetected with these tests.

#### 4.1.4. Stool DNA testing

This non-invasive method tests for the presence of molecular debris and occult blood in the stool samples (63). This debris might include mutant DNA seen in tumor cells, like mutant KRA, p53, aberrantly methylated BMP3, NDRG4 promoters, etc. (64). Cologourd®, an FDC-approved multi-target stool test, has been shown to have higher sensitivity than FIT (92 and 72% respectively) but lower specificity (92 and 74% respectively), in a study that tested both on nearly 10,000 patients, using colonoscopy as reference. It also had a low detection rate for large advanced melanomas of only 42%, therefore limiting its preventive role (64).

#### 4.1.5. Computed tomography colonography

Computed tomography colonography, or CTC, provides images of the entire abdomen and pelvis, not just the colon. It uses a radiographic agent to non-invasively tag stool for digital imaging. CTC’s per-person sensitivity for adenomas below 10 mm varied between 66.7 to 93.5% in a meta-analysis evaluating its effects with colonoscopy, while its specificity values ranged from 96.0 to 97.9% (65).

#### 4.1.6. Double-contrast barium enema

Double-contrast barium enema or DCBE is performed without any use of sedative and involves the injecting of air and rectal contrast and is therefore an unpleasant experience for the patient. But it can evaluate the entire colon for any abnormality (51). A study between DCBE and colonoscopy showed that DCBE detected only 32% of polyps less than 5 mm, 53% of polyps 6 to 10 mm, and only 48% of those greater than 1 cm (66).

#### 4.1.7. Serological tests

A blood-based detection test, or liquid biopsies, checks for DNA markers floating in blood. The presence of methylated septin 9 in plasma has been assessed in many studies (67). According to a meta-analysis study that was based on 25 research articles, the SEPT9 assay is only better than the FIT in the symptomatic group (68). The test’s current commercially available iteration has a sensitivity for advanced neoplasia and CRC of 25 and 68%, respectively, with a specificity of 79% (69, 70) (Table 2).

**Table 2:** Summary of detection techniques used for CRC detection based on cost-effectiveness.

Techniques used	Sensitivity	Specificity	Cost effectiveness	References
Colonoscopy	95%	88–98%	Higher cost when compared to other methods.	(54)
Sigmoidoscopy	95%	87%	More affordable than a colonoscopy	(59)
Colon Capsule Endoscopy	76.7%	90.7%	More expensive than a colonoscopy	(12)
g-FOBT	96–98%	50–75%	More affordable than a colonoscopy	(12)
FITs	94%	74%	More affordable than a colonoscopy	(70)
stool DNA testing	85%	93%	More expensive than a FITs	(64)
Computed tomography colonography	66.7–93.5%	96.0–97.9%	More affordable than a colonoscopy.	(65)
DCBE	80%	95%	Almost same as colonoscopy	(66)
Serological tests	68%	79%	More affordable than a colonoscopy	(69, 70)

### 4.2. Current advancements in biomarkers for early detection of colorectal cancer

The need for more specific and sensitive biomarkers to detect CRC arises from the fact that CRC is one of the top four most prevalent cancers worldwide (71) with a high mortality rate. The current non-invasive techniques used for screening, for example, are not very sensitive to the earlier stages of cancer and may miss any pre-cancerous lesions and polyps. According to Imperiale et al. (72) “In asymptomatic persons at average risk for colorectal cancer, multitarget stool DNA testing detected significantly more cancers than did FIT but had more false positive results” (73). The finding further establishes the need for more sensitive biomarkers along with the already used screening techniques. The emergence of gene expression analysis along with transcriptome studies has allowed scientists to categorize CRC into subtypes for developing a better understanding of the disease and for devising better treatment strategies based on the subtype of CRC a patient may have. (74)Maida et al. (Maida et al., 2017) performed Molecular sub-typing of colorectal cancer, dividing it into 4 major subtypes: CMS1, CMS2, CMS3, and CMS4. This analysis has also elucidated new biomarkers. Similarly, Multi-Omics studies analyzing large amounts of data on the structure and function of several biological molecules in their totality have led to a better understanding of multifaceted and complex diseases like cancer. The omics studies including genomics, transcriptomics, proteomics, metabolomics, glycomics, etc. have revealed many new promising biomarkers for CRC. These biomarkers include several different kinds of molecules which may be DNA–RNA-based, protein-based, metabolite-based, or even volatile substances found in a patient’s breath. They can be detected utilizing techniques like genomic analysis, mutation analysis using hybridization arrays, micro-arrays, bioinformatics analysis, mass spectroscopy, Gas- Chromatography MS, Gel electrophoresis, etc. (9).

## 5. Effect of NSAIDS on the gastrointestinal system

PGs increase mucus production and PGI2 and PGE2 have a vasodilator effect on the vasculature of the gastric mucosa and reduce gastric acid output. On the other side, NSAIDs could prevent the effects of PG on the gastrointestinal tract. Mucosal proliferation, HCO_3_ secretion, and mucin synthesis are all inhibited by this action. NSAIDs can damage the gastrointestinal tract by impairing this function, which can lead to stomach problems (75). Gastric hypermotility results from NSAID usage that inhibits COX-1. Although the exact process is unknown, it is possible that tissue hypoxia and microvascular damage arise from high-amplitude, limited blood flow. There is some evidence that NSAID usage may lower the chances of GI malignancies, including gastric, pancreatic, and colorectal cancers, in contrast to the acute effects of NSAIDs on the GI tract (76). For instance, multiple studies have discovered that NSAIDs without aspirin are linked to a lower risk of gastric cancer (77) and, in the case of celecoxib, a higher rate of per-cancerous gastric lesions regressing when compared to placebo. To identify these possibly beneficial effects more fully, more research is nonetheless required (78).

### 5.1. Effect of NSAIDS and relation between cancer and inflammation

Acute inflammation, also known as resolved inflammation, is a self-limiting adaptive host defense mechanism that brings the body back to a state of homeostasis. However, persistent, unchecked, or unresolved inflammation can result in a number of diseases, including cancer. Nonsteroidal anti-inflammatory drugs (NSAIDs), like aspirin, lower the risk and death from several malignancies, which is significant evidence that connects inflammation and cancer. Clinical studies using COX-2 inhibitors for cancer prevention or therapy were justified by the overexpression of COX-2 in the colon and many other malignancies. NSAIDs, on the other hand, do not need COX-2 to prevent cancer (79).

Since ancient times, it has been understood that the primary reaction to damage is “Inflammation.” Hippocrates, a Greek physician, may have been the first to view inflammation as the start of a healing process and used terms like *erysipelas* and *edema* to characterize its symptoms (80). The body’s reaction to an exposure, such as an infection or an injury, is inflammation. NSAIDs have been identified as the prototype chemopreventive drugs against several types of cancer by more than 30 epidemiological investigations that combined reported findings on more than one million participants. NSAIDs can affect the microenvironment of tumors by slowing cell migration, boosting apoptosis, and decreasing chemosensitivity. Targeting the molecules (COX-2 cyclooxygenase 2, NF-kB, VEGF) involved in the inflammatory process might offer a useful technique for cancer prevention and therapy since they can predispose to tumors (81). Several NSAIDs like aspirin, celecoxib, piroxicam have shown preventive effects on inflammation in colorectal cancer. Colorectal cancer-related prevention by NSAIDs mostly works by acting on the pathway of the eicosanoids (82). NSAIDs have been shown in the past to have anti-tumor effectiveness, less toxicity, and non-specific side effects than those caused by conventional chemotherapy. They were also able to limit tumor growth by causing changes in the inflammatory environment of the tumor (83). NSAIDs have demonstrated chemoprotective and anti-inflammatory effects on inflammations associated with tumors. The fact that COXIB has more notable protective advantages than non-selective NSAIDs against a variety of malignancies is associated with a larger reduction in the risk of cancer (84, 85).

## 6. Role of NSAIDs in colorectal cancer chemoprevention

Cyclooxygenase (COX)-dependent and independent pathways participate in anti-tumorigenesis, albeit their mechanisms are not completely known (86). The primary anticancer action of NSAIDs is assumed to be a COX-2 inhibition-mediated suppression of prostaglandin E2 production, which reduces tumor cell proliferation, angiogenesis, and enhances apoptosis. Various signal transduction pathways like nuclear factor-kappa B, NF-κB, have been proven as COX-independent NSAID-induced effects, despite the fact that many of the anticancer mechanisms of NSAIDs are described as COX-dependent. Numerous studies have been conducted on the relationship between expression of COX and colorectal cancer, including prognostic variables and potential chemo-preventive drugs (87).

### 6.1. Mechanism of anti-cancer activity of NSAIDs

#### 6.1.1. COX dependent pathway

COXs are regulators that have critical roles in carcinogenesis, angiogenesis, and inflammation. COXs found on luminal side of the ER (endoplasmic reticulum) are connected with the nuclear envelope and have three isoforms: COX 1, COX 2, and COX 3 (58, 88). The pharmacological basis for anti-inflammatory activity of NSAIDs is believed to be the inhibition of COX 1 and COX 2 enzymes, which catalyse conversion of arachidonic acid into prostaglandin H2, a precursor for the formation of prostacyclins, thromboxanes and prostaglandins. These eicosanoids have been associated to pain, fever, and inflammation. Moreover, they protect stomach and gut lining from harmful impact of the acid, stimulate blood clotting by activating blood platelets, and control kidney function. COX 2 is triggered by inflammatory stimuli, whereas on the other hand COX 1 is constitutively expressed in several tissues and has a significant role in the tissue homeostasis (89).

Several molecules linked to inflammatory and malignant processes have their gene transcription and protein synthesis regulated by aspirin and NSAIDs (90). The ability of NSAIDs and aspirin to decrease COX expression and downstream signals, that are essential for CRC cell diffusion, survival proliferation, allows for differentiation of these actions. Arachidonic acid is transformed into prostaglandin G2 by cox enzymes which is an unstable intermediate that is quickly degraded into PGH2. After that, PGH2 is transformed in a number of PGs with comparable structural properties, such as Thromboxane (TX) A2, PGD2, PGF2, PGI2, and PGE2 (91). Despite the fact that research in experimental CRC models has shown that COX 1 may promote cancer growth, in mammalian tissues, COX 1 is expressed constitutively, and PGs synthesized from COX 1 are required for physiological functions (56).

On the other hand, COX 2 is activated in various cell types by tumor promoters, growth factors, and inflammatory cytokines (92). In 80-90% of carcinomas and 40–50% of human colorectal adenoma cancers, COX-2 expression is elevated, which increases PG synthesis (93, 94). Platelet-derived growth factor, matrix metalloproteinases, and vascular endothelial growth factor are all vital for the genesis, development, and advancement of tumors. COX 2 stimulates the synthesis of these molecules. Additionally, COX-2 restricts the development of immune cells with antineoplastic activity and controls the production of proteins that are both pro- and anti-apoptotic (61, 95).

Aspirin is the NSAID which has the ability to permanently suppress COX 1 and COX 2 action. On antiplatelet therapeutic levels of 75-100 mg daily, aspirin is 100-fold more effective than monocyte COX-2 in suppressing platelet COX-1 (96). Platelet activation in CRC patient stimulates the generation of proteolytic enzymes and chemokines which promote metastasis, angiogenesis and cancer cell proliferation (97). Activated platelets may potentially contribute to COX 2 overexpression in CRC by producing TGF, IL-1 and platelet-derived growth factor (97). Aspirin’s anti-platelet activity may thus be responsible for some of its anti-tumorigenic actions. Aspirin and other NSAID suppression of PGE2 and COX 2 synthesis may depend on modulating a variety of signals which also includes sphingosine-1-phosphate (S1-P) synthesis suppression and activation of NAG-1, a gene induced by NSAID.

#### 6.1.2. COX independent pathway

COX inhibition does not account for all of the NSAID-mediated anticancer effects. In fact, not all NSAIDs which are COX inhibiting possess anticancer effects and reactivating COX does not release the CRC cells from the arrested cell growth induced by NSAIDs. Additionally, CRC cells deficient in COX experience NSAID-induced apoptosis and growth inhibition (98).

##### 6.1.2.1. NF-κB activation

Different subunits of the NF-κB family are regulated by NSAIDs in Colorectal cancer and can combine to produce homodimers and heterodimers. Among these, the binding of the RelA (p65) and p50 heterodimer occurs in an inactive state in cytoplasm with the help of I-kappaB (IκB) inhibitor protein. The translocation of this heterodimer to nucleus occurs in response to the activating stimuli which leads to phosphorylation of IκB with its subsequent degradation by proteasome. Translocated p50/RelA heterodimer controls the transcription of various genes (99). Reduced NF-κB transcriptional activity is resulted from the nucleolar sequestration of RelA induced by a dose of 5–10 mM of aspirin in cultured CRC cells (100). A dose of 50 μM of Sulindac sulfide limits the HCT-116 invasion of cells by inhibiting transcription (mediated by NF-κB) of some particular microRNAs like miR 9, miR 17, miR 21 which regulates gene expression implicated in metastasis and tumor cell invasion (101).

##### 6.1.2.2. Wnt/β-catenin pathway

**Figure 2:**
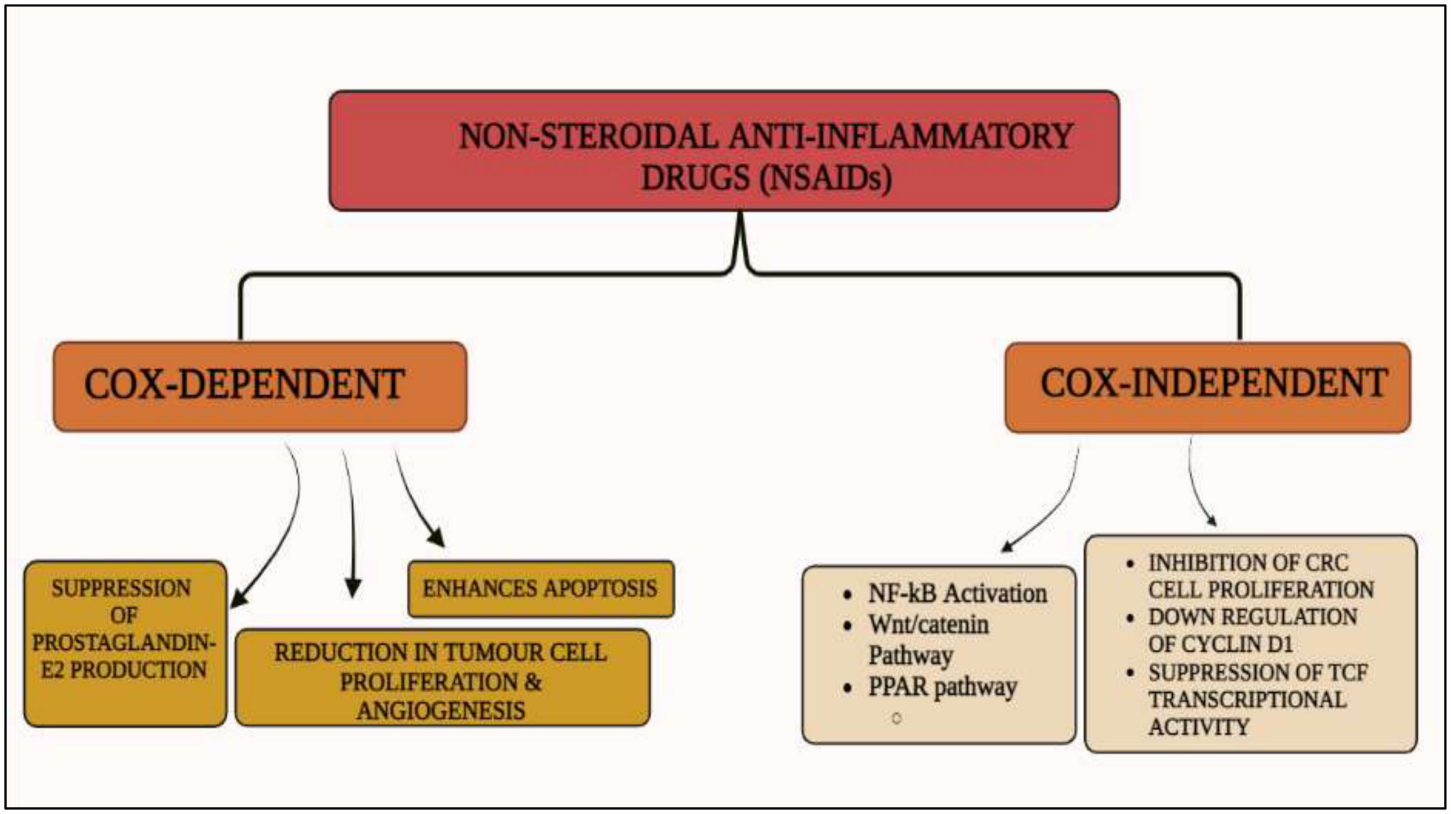
Cox-dependent and independent mechanism overview associated with NSAIDs. Cyclooxygenase dependent and independent pathways play a significant role in anti-tumorigenesis. The major anticancer action of NSAIDs is thought to be COX 2 suppression mediated decrease of prostaglandin E2 synthesis, which inhibits tumor cell proliferation and angiogenesis while increasing apoptosis.

**Figure 3:**
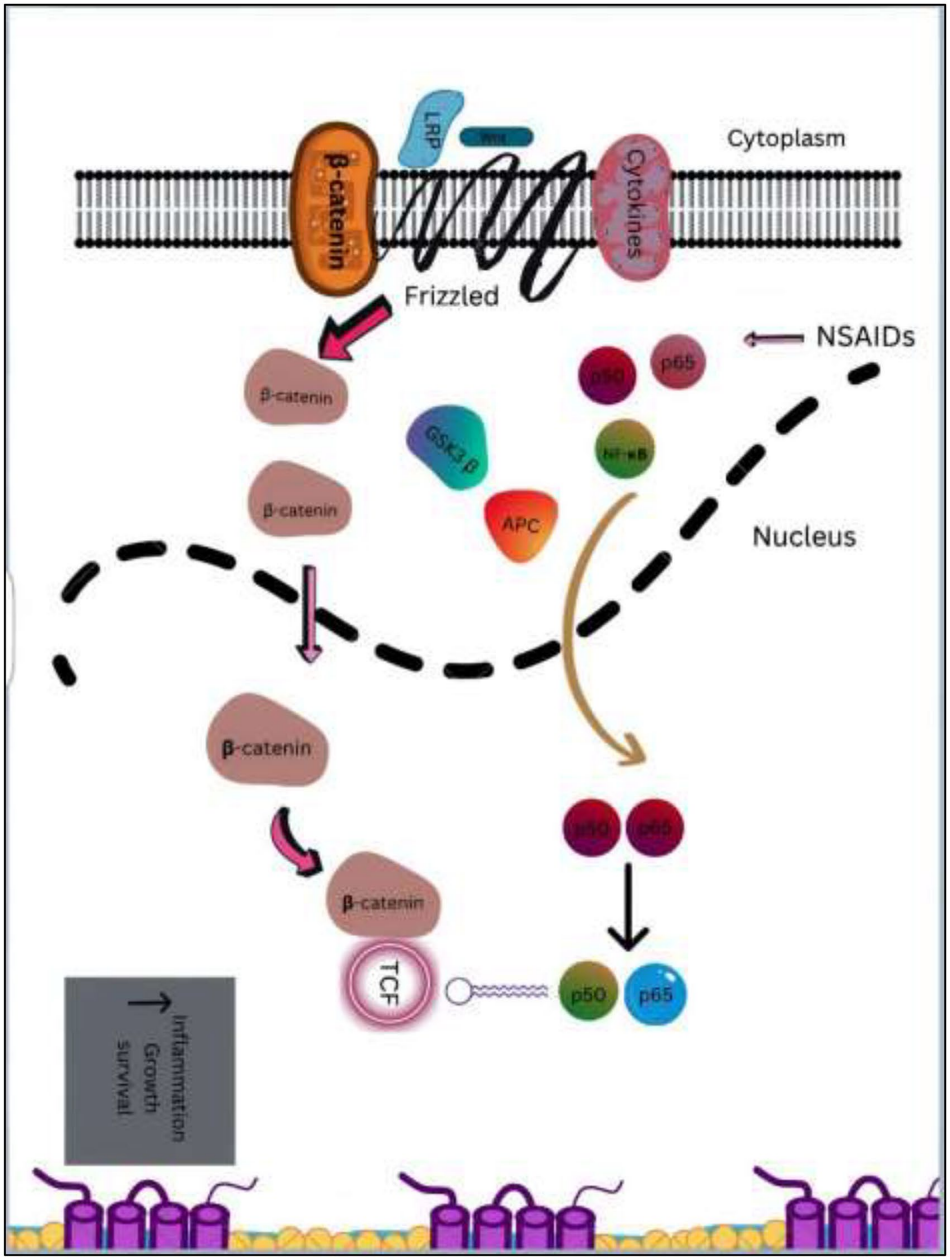
An overview of downstream targets in colorectal cancer & NF-κB and β-catenin/Wnt pathways. Catenin accumulates as a result of APC gene or activating mutations in the β-catenin, which leads to the formation of complex with the TCF/LEF transcription factors. TCF can interact with extra to stimulate the transcription of genes which are proliferative in the colon, including c-Myc and cyclinD1. With the release of p65, that is subsequently translocated to nucleus, inflammatory cytokines activate NF- κB, which leads to an increase in target gene transcription. NSAIDs in combination with other drugs like naproxen or sulindac targets β-catenin /Wnt and NF-κB pathways and suppresses downstream signaling.

Wnt/β-catenin pathway (Wingless and integration site growth factor (Wnt)/-catenin) is a pathway that NSAIDs can target easily, since it is active in most of the CRC cells. A protein called cytoplasmic disheveled (Dsh) protein is activated by binding of Wnt with TFR (transmembrane frizzled receptor). The glycogen synthase kinase 3 (GSK3), casein kinase 1 (CK1), protein phosphatase 2A (PP2A), axin, and Apc, make up the catenin destruction complex, which Dsh protein binds with. The ubiquitination and degradation of the destruction complex is facilitated by the phosphorylation of the cytoplasmic β-catenin while Wnt signaling being absent (102). However, a decrease in the β-catenin degredation in response to the Wnt signals is seen with the aggregation of cytoplasmic β-catenin and gradual translocation to nucleus. Therefore, gene expression that promote tumor, for example, c-jun, peroxisome proliferator-activated receptor delta, c-myc, cyclin D1, and matrilysin is stimulated by the binding of β-catenin with the family components of LEF (lymphoid enhancer factor) and TCF (T-cell factor) (103).The β-catenin phosphorylation is enhanced by 5 mM and 100 mM doses of aspirin and celecoxib which reduces its nuclear aggregation and, as a result, transcription of Wnt/-catenin target genes in colorectal cancer cells (104). A study reported more data supporting the Wnt/-catenin pathway as a target of NSAIDs in CRC chemoprevention. According to this study, a 50 μM dosage of sulindac sulphide suppresses TCF transcriptional action of Wnt/β-catenin without enhancing phosphorylation of β-catenin, hence downregulating cyclin D1, and specifically inhibiting CRC cell proliferation (105). Several types of NSAIDs and their chemical structures have been discussed under Table 3 (Figures 2, 3).

**Table 3:** A systematic representation of mechanism of action of NSAIDs along with their advantages and drawbacks.

Name of the NSAID	Chemical structure	Mechanism of action	Advantages in CRC	Drawbacks	References
Aspirin	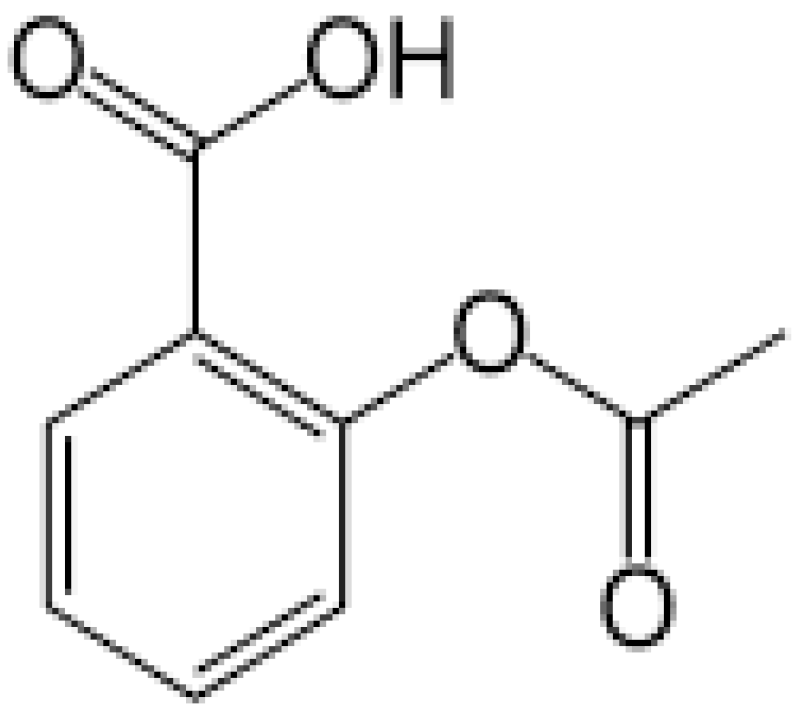	- Inhibit COX1 and COX2 in CRC tissues. - PIK3CA pathway inhibition (COX independent)	Reduces colorectal polyps and inflammation	GI bleeding	(106, 107)
Diclofenac	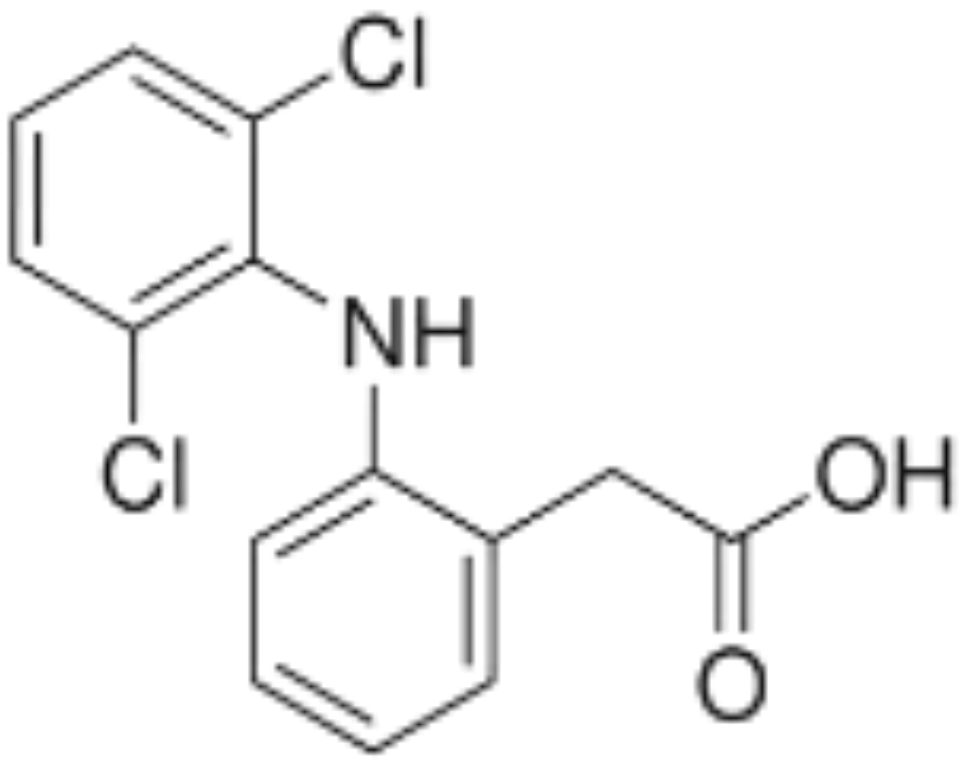	- Inhibit Wntβ catenin signaling *via* NF-kβ	Reduce inflammation	Abdominal discomfort, nausea, diarrhea	(108, 109)
Ibuprofen	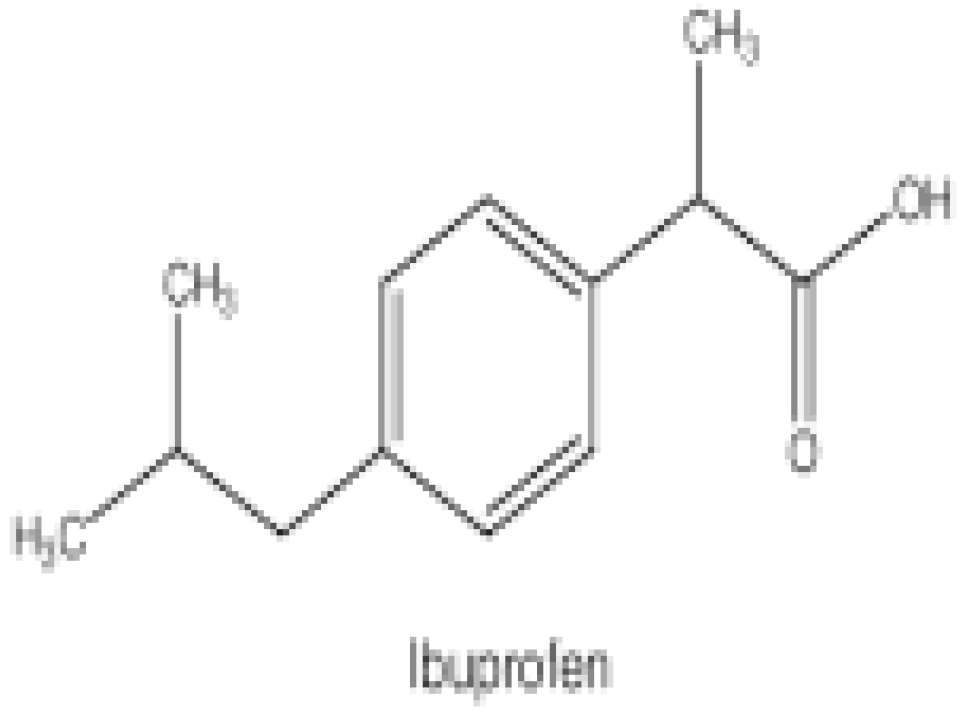	- Inhibition of MAPK, NFkβ (COX independent) - COX dependent inhibition	Reduce inflammation	GI bleeding	(110, 111)
Indomethacin	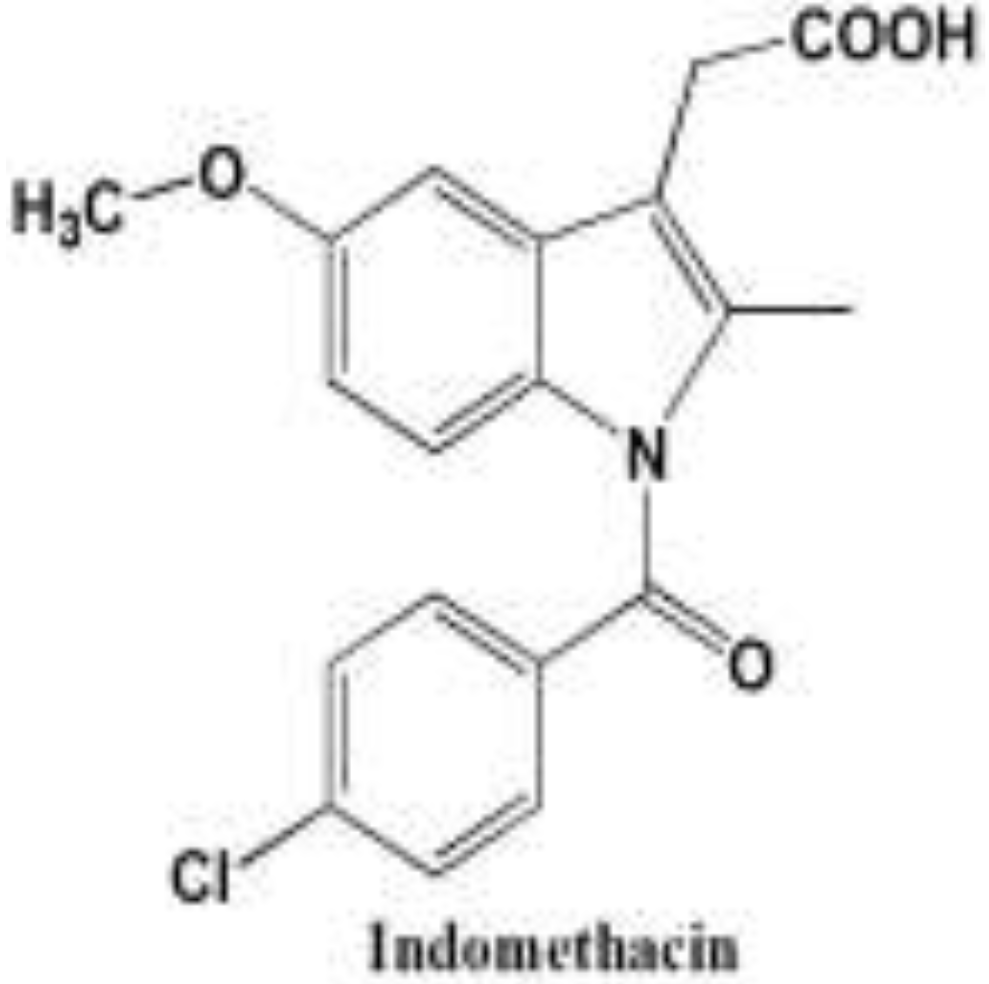	- NF-kβ, PPARδ inhibition - COX dependent inhibition	Anti-proliferative and apoptotic effects	Gastric ulceration and renal toxicity	(108, 109)
Ketorolac	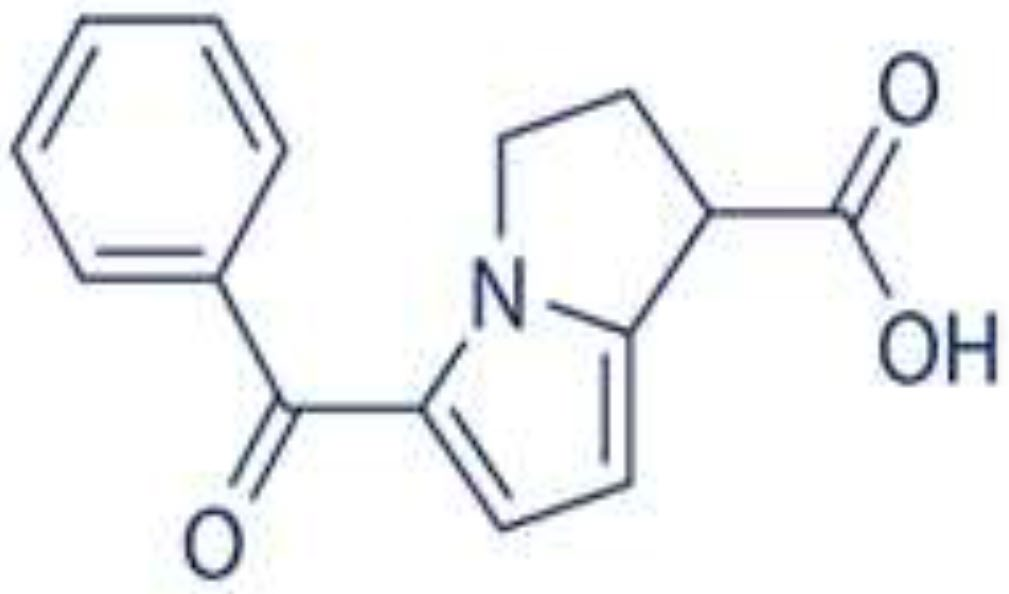	- COX1 and COX2 dependent inhibition	Anti-metastatic effects	Post surgical anastomotic leak	(112)
Oxaprozin	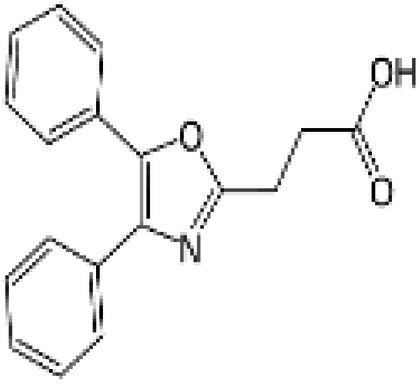	- COX1 and COX2 dependent inhibition	Anti-metastatic effects	Cardiovascular risk, GI ulceration	
Rofecoxib	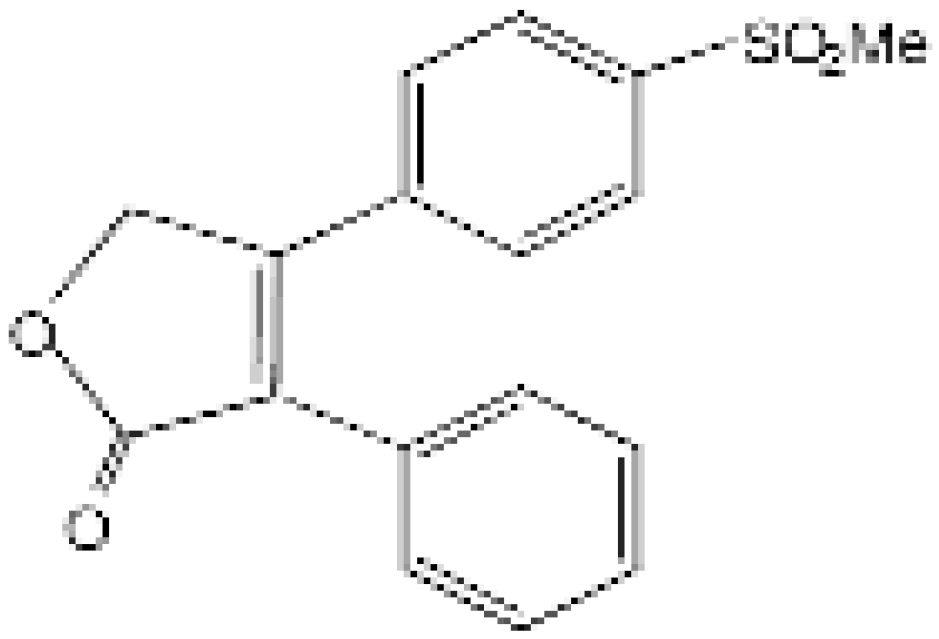	- COX dependent inhibition	Anti-inflammatory and analgesic properties	Cardiovascular risk, strokes	(110, 111)
Sulindac	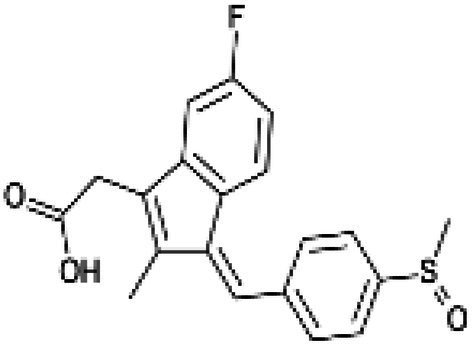	- COX dependent inhibition - Inhibition of Wnt/βcatenin pathway	Reduced colorectal polyps, anti-inflammatory roles	GI ulceration and bleeding	(106, 107)
Celecoxib	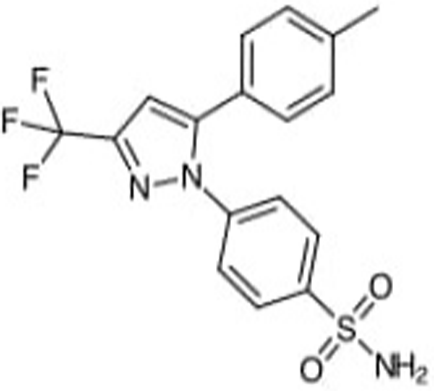	- COX2 inhibition - MAPK pathway inhibition	Decreased recurrence of colorectal adenoma	GI bleeding, ulceration and cardiovascular risk	(106, 107)

### 6.2. Combined use of statins and NSAIDS for synergistic effect in CRC-chemoprevention

The drugs which lower cholesterol, also known as statins are made of tiny molecules called 3-hydroxy-3-methyl glutaryl coenzyme-A (HMG-coA) reductase inhibitors. Since statins show anti-carcinogenic characteristics in several *in vitro* and *in vivo* preclinical tests, there is a great interest in finding out how they might be used in cancer chemoprevention. Statin use may offer some preventive benefits against total cancer risk, according to some observational human research, but not others (113).

Statins are routinely used to reduce cholesterol and NSAIDs are mainly used to treat inflammation. Recent studies have focused on their potential function as cancer chemo-preventive drugs. Human studies have not shown solid data on the protective benefits of statins against various malignancies, although NSAIDs have yielded more compelling results for cancer prevention, particularly in CRC. Combining statins with NSAIDs may induce synergy and result in a reduction in the doses needed for each agent, which is a potential technique for improving cancer prevention effectiveness. This method is of particular importance for the prospective long-term utilization of low dosages of NSAIDs and statins for cancer chemoprevention. Significantly, colorectal cancer chemo-preventive studies have shown elevated possibility for gastrointestinal and cardiovascular adverse effects linked to NSAID usage. A growing body of research has conclusively shown that NSAIDs help prevent cancer, particularly colorectal cancer. Because of the potential elevated risk of severe cardiovascular and gastrointestinal side effects, relatively high dose needed to produce the observed chemo-preventive benefit in human studies can dissuade the long-term usage of NSAIDs alone for cancer prevention (114). Emerging research suggests that combining cancer chemo-preventive drugs, NSAIDs with distinct mechanisms of action may result in synergistic interactions, which might result in far higher anti-carcinogenesis benefits than each chemo-preventive agent could independently. NSAIDs have demonstrated synergistic effect in various other *in vitro* studies when treated with other therapeutic agents for example EGFR family inhibitors, statins, TRAIL receptor ligands, and PPARg ligands (115).

The combined use of statins and NSAIDs is particularly intriguing for cancer prevention. Atorvastatin is an example of the drug that was prescribed most in the year 2006 in US. In a significant experiment, to examine the results in individuals with coronary artery disease, pravastatin usage was found to be linked to a 43% decrease in several newly detected instances of colon cancer. Notably, 83% of individuals in both placebo and pravastatin groups received aspirin every day, implying that interaction between aspirin and pravastatin may have an improved protective impact (116).

The effects of statins and aspirin on risk of CRC were studied in a population-based case control research (117). This study comprised 612 controls and 537 patients with CRC cases that had been histologically proven. Frequent use of aspirin at a low dosage level was linked to a moderate reduced risk for CRC, whereas frequent use of statins, primarily simvastatin and atorvastatin, was linked to a stronger risk reduction. The most intriguing finding was that taking statins and lower dose of aspirin together for 5 years or more was linked to 62% risk of risk in CRC.

Utilizing the AOM rat model, effectiveness of celecoxib, aspirin, and atorvastatin against colon carcinogenesis when given separately on high dosage levels and when combined at low dosage levels (118). In comparison to single high doses of atorvastatin given at 150 ppm or celecoxib given at 600 ppm, the combination of 100 ppm atorvastatin and 300 ppm celecoxib reduced the prevalence and multiplicity of adenocarcinomas. Accordingly, low-dose combination of atorvastatin and aspirin significantly inhibited the prevalance and multiplicity of adenocarcinoma when compared to higher doses of each treatment alone. The effects of celecoxib and atorvastatin was examined on growth of adenomatous polyps in intestines in a different experiment utilizing the ApcMin/+ mouse model. Combining atorvastatin and celecoxib at 100 ppm and 300 ppm, respectively, was found to completely suppress colonic adenomatous polyps and reduce adenomatous polyps in small intestines by 86%. However, these effects were more potent than those brought on by either celecoxib or atorvastatin treatment administered separately (119). Together, these findings certainly showed that statin/NSAID combination regimens significantly increased the effectiveness of either type of agent administered alone in preventing cancer. This strongly supports the use of the statin/NSAID combination as a promising method for cancer chemoprevention.

#### 6.2.1. Pathway involved

The pathways through which statins and nonsteroidal anti-inflammatory drugs (NSAIDs) limit cancer cell proliferation, induce apoptosis, and block other procarcinogenic processes are not completely known. Examples of celecoxib and atorvastatin were selected to briefly describe the potential mechanism of statins and NSAIDS as cancer chemo-preventive medications. By inhibiting HMG-CoA reductase, the rate-limiting enzyme in the mevalonate pathway, statins reduce the formation of isoprenoids such geranylgeranylpyrophosphate (GGPP) and farnesylpyrophosphate (FPP; FPP). These isoprenoids are necessary for the isoprenylation, membrane localization, and subsequent activation of a number of signaling proteins, such as Ras, Rho, and Rac. In contrast to GGPP, which can stop the apoptosis that statins cause in cancer cells, add-back assays showed that FPP had little to no protective benefits (120). These results demonstrated that GGPP had a more significant contribution to statin-induced effects than FPP. Studies have shown that geranylgeranylated Rho proteins play a part in the effects that statins induce, whereas the findings on farnesylated Ras have been contentious (121).

The specific mechanism by which statins and NSAIDs operate synergistically to create improved anti-carcinogenic effects remains largely unknown. A study was carried out on colon cancer HCT 29 and HCT116 cells. The mode of action was studied, and a strong synergistic effect was observed (122). Cell cycle arrest in the G0/G1 phase was brought on by the atorvastatin/celecoxib combination therapy for 24 h, and this effect was substantially stronger than those brought on by atorvastatin or celecoxib alone. These results are in line with those from animal studies, which showed that atorvastatin and celecoxib combination therapies reduced proliferative index and elevated apoptotic index in tumor tissues. Other studies in cancer cells demonstrated increased apoptosis with statin and NSAID co-treatments (123).

**Figure 4:**
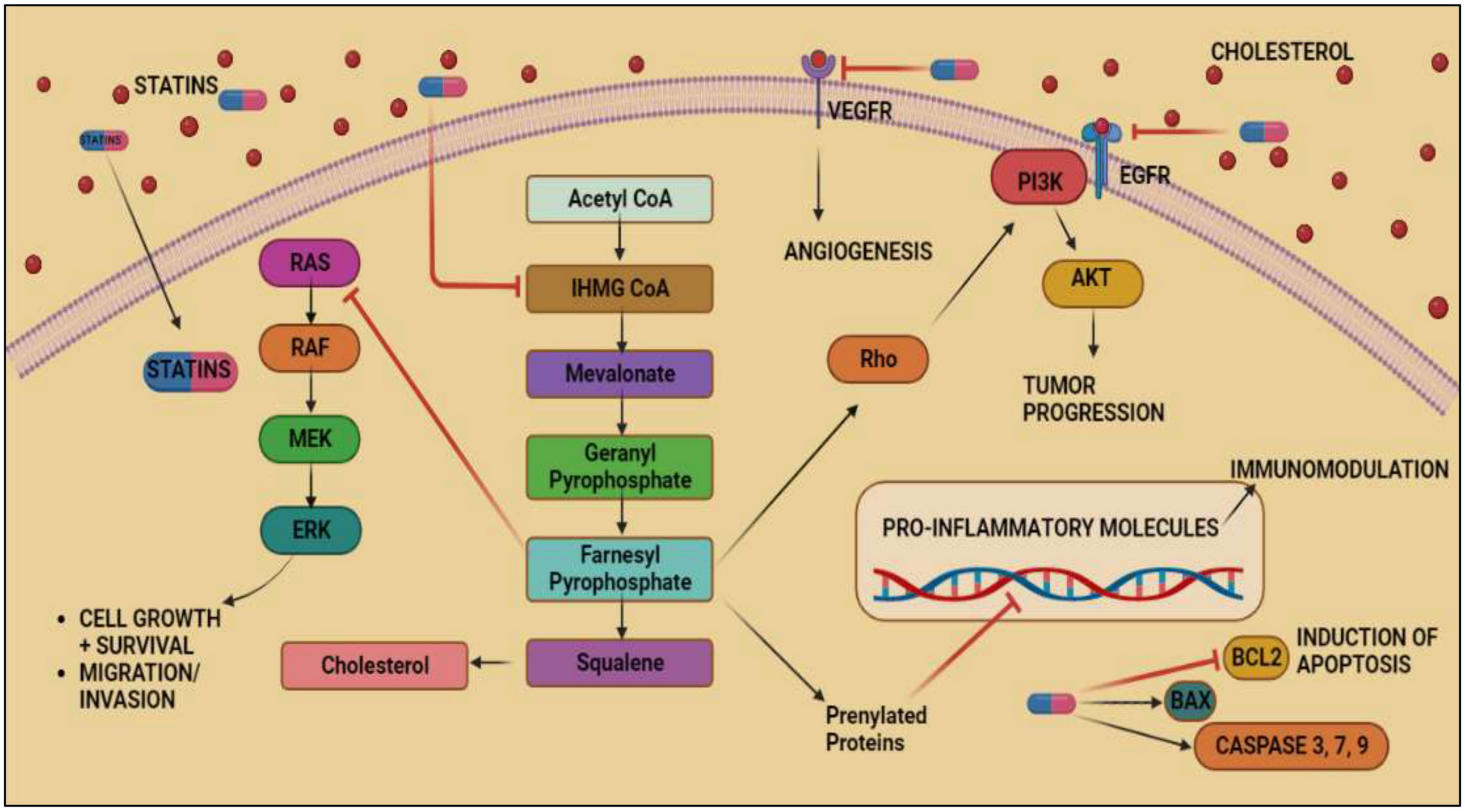
Anticancer effects exerted by Statins by inhibiting mevalonate pathway. Acetyl-CoA, the byproduct of glycolysis, is converted into mevalonate, IPP, GPP, FPP, GGPP, and cholesterol through a series of enzymatic processes that make up the mevalonate pathway. FPP and GGPP may both be supplemented to proteins post-translationally, particularly minor monomeric GTPases such as Ras predominantly part of MAPK/ERK pathway responsible for inducing VEGF expression in colorectal cancer. The inhibitory effect of FPP on MAPK/ERK pathway and inhibition of mevalonate pathway by statins causes tumor cell death and prevents migration of tumor cells. Statins shows its inhibitory effect on VEGFR and EGFR thus, inhibiting angiogenesis and tumor progression in cancer. It also inhibits BCL2 and induces aoptosis of cancerous cells.

**Figure 5:**
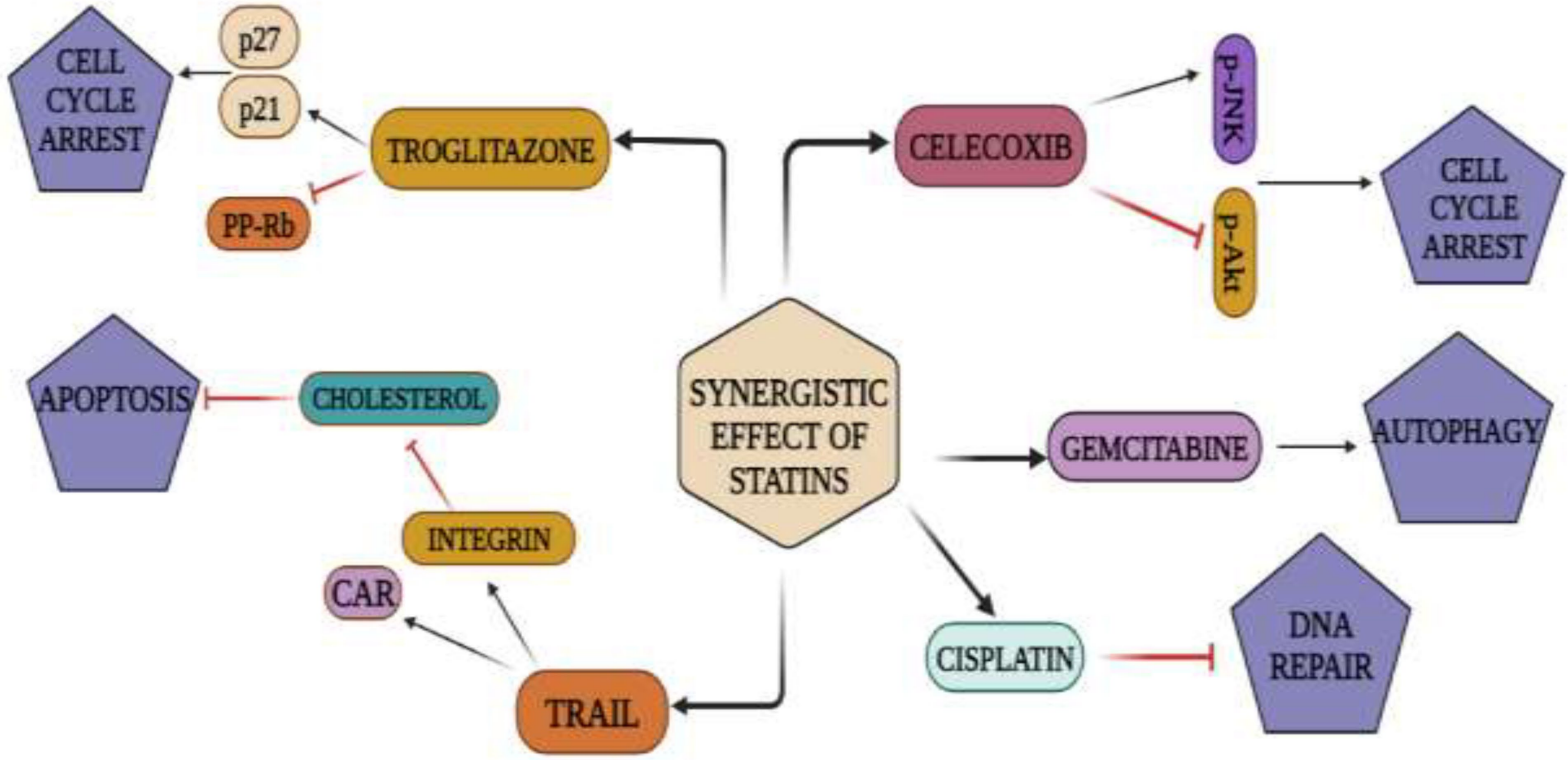
Synergistic action of statins and NSAIDs: Statins repress and activate signaling cascades that result in cell-cycle arrest, cell death, apoptosis, and autophagy when used with anti-cancer medications such TRAIL, troglitazone, celecoxib, gemcitabine, cisplatin.

According to research, atorvastatin lowers the level of membrane bound RhoA, probably by isoprenylation inhibition and its impact is greatly boosted when combined with celecoxib (124). This may inhibit RhoA’s carcinogenic actions, which have been linked to cell cycle progression, enhanced tumor invasiveness, and metastasis (125). The suppression of RhoA’s membrane attachment is one potential method by which the combination of atorvastatin and celecoxib might cause cell cycle arrest. This can cause disruption of RhoA’s negative control on both p21Cip1/Waf1 and p27Kip1 and that may raise the levels of these two CDK inhibitors (126). Unlike RhoA, the combination of atorvastatin and celecoxib raised the membrane-bound RhoB by an unknown mechanism. Due to the potential tumor-suppressing action of RhoB, the enhanced membrane association of RhoB may contribute to the inhibitory effects of atorvastatin/celecoxib combo on cancer cell proliferation (127). Celecoxib was discovered to strongly synergize with atorvastatin to abolish phosphorylation of Akt in colon cancer cells, even at low doses when little or no inhibition on Akt was shown on its own (128). The same treatments decreased Akt’s upstream kinases, PDK1 and PI3K, phosphorylation levels. Furthermore, by reducing PTEN’s phosphorylation at Ser380, the combination therapy may have elevated PTEN activity. In colon cancer cells treated with celecoxib or atorvastatin alone, all of these effects were either completely absent or markedly diminished. The apoptosis brought on by the combination of atorvastatin and celecoxib therapy may be significantly influenced by the suppression of the Akt pathway (128). It is crucial to note that neither of the two human colon cancer cell lines used had enough COX-2 expression. HCT11 cells lack the enzymatically inactive COX-2 protein that HT29 cells express (129). As a result, the effects of celecoxib and its combination with atorvastatin reported in this study were COX-2 activity independent. Findings on the combined treatment of licofelone (a dual inhibitor of COX-1 and 2) as well as 5-lipoxygenase, and atorvastatin did not show a significant synergy in inhibiting HCT116 cellular proliferation. More research is required to validate the involvement of COX-2 in the statin/NSAID combined treatment (Figures 4, 5).

#### 6.2.2. The Nanoformulation of NSAIDs for CRC chemoprevention

Nanotechnology encompasses a wide range of novel and extraordinary nanomaterials with diagnostic and therapeutic potential. Carbon nanotubes, liposomes, dendrimers, gold nanoparticles, silica nanoparticles, and other nanomaterials are employed in colorectal cancer diagnosis and therapeutic delivery. Various drugs loaded on gold and silica nanoparticles are engaged in the death of CRC cells by targeted delivery of anticancer medications to cancer cells. With technological innovation, new approaches incorporating the utilization of nanotechnology have paved the way for the manufacture of nanomaterials capable of treating CRC cancer as well as other tumor types. These approaches have also aided in the identification and screening of CRC. The use of nanotechnology in CRC is crucial for the development of tailored drug delivery systems, the early detection of malignant tumors (which are nanomaterial-based), and several other improved therapeutic approaches. Regarding the present progress of nanotechnologies in the treatment of CRC, it has gained global attention due to its capacity to enhance screening techniques as well as diagnosis and therapy. Nanoparticles have been shown to increase current information on biochemical and physiological principles underlying a few diseases and their therapies. Nanoparticles have shown improved performance in few techniques like PET (positron emission tomography) and MRI (magnetic resonance imaging) with the respective use of radioisotope chelator-free nanoparticles in PET and iron-oxide based nanoparticles in MRI.

Due to their small size, remarkable sensitivity, and unique chemical constitution, nanoparticles are ideal contrast agents and are frequently employed in the treatment of cancer. When used therapeutically, it enhances the aggregation and discharge of pharmacologically active substances at the diseased site, increasing therapeutic efficacy and minimizing adverse toxic side effects. Additionally, NPs which have been recently developed have the capacity to combine diagnostic and therapeutic compounds into a single nanoparticle that is simple to employ for theranostic applications. Theranostic nanoparticles (NPs) may also be used in individualized nanomedicine-based therapeutics, according to studies. To develop an efficient treatment for colorectal cancer, new technologies for detecting proteins, genes, and other components in an individual’s cancer should be devised. Anti-angiogenesis therapy is an alternative for CRC treatment in addition to EGFR inhibitor therapy. The most prevalent negative effects of targeted treatment are appearance of upper body and facial rashes. Poor drug responsiveness to chemotherapy while treating CRC is commonly observed, and this may be largely because of the development of multidrug resistance in tumor cells. Nanomedicine is believed to be a current method to improve the prognosis and treatment for CRC patients in order to combat multidrug resistance.

Numerous significant nanotechnological applications in cancer biology have been established, including early cancer screening and diagnosis as well as the development of novel therapy modalities that cannot be achieved with the currently available conventional technologies. In fact, particles bearing nano sizes of various forms and constitution have evolved as crucial and promising innovative tools for colorectal cancer screening, diagnostics, and treatments.

Different nano-formulations have been developed throughout the years to enhance curcumin delivery to cancer cells or tissues. Nano-formulations are generally utilized to improve solubility of curcumin in water and provide more constant curcumin administration (107, 130). Also, Curcumin nano-formulations treating tumors should ideally have increased anticancer efficacy when compared to curcumin alone and be harmless to normal cells.

Various studies have reported the documentation of curcumin nano-formulation for colorectal cancer treatment. The studies involve the use of polymeric nanoparticles, nano gels, liposomes, gold nano particles, cyclodextrins, solid lipid nanoparticles etc. Even though several nano formulations are through clinical testing, the number of nano formulations employed in CRC clinical trials is restricted. With the improvement in the designing of nano devices, nanomedicine has demonstrated its effectiveness in transforming the treatment and diagnosis of cancer. The drug-encapsulation methods that are on the nanoscale are particularly effective in passively retaining additional drug-loaded NPs close to cancer cells. These tactics have aided in the establishment of the subsequent generation of anticancer nanomedicine.

The main purpose of NSAIDs is to prevent colon cancer. The epidemiological studies show that aspirin is the most promising NSAID of all the reported ones. Whereas the prevention of colon cancer by aspirin either alone or in combination has been demonstrated, nano encapsulation of aspirin can increase its effectiveness at a lower dose. A study conducted on seven-week-old male Sprague Dawley rats which were treated with azoxymethane revealed the chemo protective impact of calcium, folic acid, and aspirin. It was discovered that this combination was 1.7-fold more effective than their unmodified complement routine (111, 131). The clinical uses of another NSAID, known as celecoxib in context of chemo preventive activity has been widely explored. The preparation of celecoxib polymeric Nanoparticles with ethyl cellulose, lipid hybrid nanoparticles, sodium casein ate bile salt, and micro emulsions improved the drug’s bioavailability and permitted a reduction in dosage, crystallization, and associated toxicity. Phytochemicals are naturally derived plant-based compounds that are widely explored as possible chemo preventive agents and they are non-toxic and have pleiotropic properties. Curcumin has demonstrated effective chemoprotective effects in colon and intestine cancer, although it exhibits limited absorption, minimal solubility in water, and poor bioavailability. To address the issue, nano capsules of curcumin whey protein were produced, which not only demonstrated >70% discharge after 48 h but also increased bioavailability and cell internalization. In subsequent studies, it was discovered that encapsulating curcumin using polymeric nano carriers improved its solubility and the treatment group receiving curcumin nanoparticles demonstrated fewer structural abnormalities, a significant decrease in tumors, and beta-catenin levels than the group receiving curcumin alone.

In the future, this knowledge might be utilized to generate new approaches for the continued development of nanotechnology to upgrade existing medications and produce newer therapeutics.

### 6.3. NSAIDs – Dosage and duration and their therapeutic effects

Studies on the detection of colorectal cancer and its prevention are currently an expanding area of clinical oncology because it is one of the most prevalent tumors in the world. An analysis of randomized controlled, double blinded clinical studies including a few NSAIDs such as aspirin, sulindac, and celecoxib and colorectal cancer chemoprevention was done for this study. People taking NSAIDs had a decreased incidence of CRC, which points to the medications sustained chemo-preventive effects in both per-clinical and clinical studies. This advanced method of treating colorectal cancer could make it less fatal and more manageable. Clinical trials have examined and analyzed different NSAIDs for their proper dosage, duration, and therapeutic effects on CRC chemoprevention (110, 132, 133). Evidence from these clinical trials determined the extent of their chemopreventiveness in CRC. Seven trials on the use of aspirin in monotherapy, polytherapy with folic acid or eicosapentanoic acid for the prevention of CRC has been completed to date. For aspirin one such study involved the people with a history of CRC and not the ones with FAP or HNPCC. Patients had to wait for at least 5 years following tumor removal before experiencing a relapse to be eligible for carrying out colorectal adenoma prevention study (CAPS). It was found that the groups receiving aspirin 325 mg per day for 3 years had reduced average number of adenomas recurrence by 35% (134). Similar encouraging results were reached in the Asian population in the clinical trials with ASA 100 mg/day for 2 years, which involved participants with adenoma and a history of colon cancer (135). The Rothwell team also looked at if there was any weight or height dependence and how aspirin affected the risk of colon cancer over the next 20 years. In people weighing 70 kg or more, they found that 75-100 mg of aspirin used once day was ineffective at avoiding cardiovascular events, sudden cardiac death, or cancer, especially in those who smoked or took enteric-coated forms, suggesting that its dosage is too low for treatment (136). Sulindac was the subject of a further double-blind, placebo-controlled investigation in FAP patients. It was discovered that standard sulindac doses did not prevent adenomas from developing in younger patients with FAP (137) despite the fact that the number of scientific experiments with sulindac was significantly lower and was too small to be trusted. They were either given 75 or 150 mg orally twice a day of sulindac or identically looking placebo tablets for 48 months. Contrarily, celecoxib has a proven track record of protecting patients who have previously experienced sporadic colorectal adenomas from developing the condition again. Over 1,500 patients participated in the PreSAP (prevention of sporadic adenomatous polyps) and APC (adenoma prevention with celecoxib) trails. Both studies findings-one evaluating celecoxib at a daily dose of 400 mg for 3 years and the other evaluating daily doses of 400 and 800 mg are in an agreement with each other. Celecoxib’s effectiveness in preventing adenoma recurrence improves with dosage (138, 139). Celecoxib’s effectiveness in treating various tumor types when administered in conjunction with cystostatic medicines or monoclonal antibodies such as gemcitabine, cisplatin, fluorouracil, or cyclophosphamide has been studied. Moreover, in studies involving certain patient population, rofecoxib has shown to have a lower incidence of adenoma recurrence (58). Usually non aspirin NSAIDs use is associated with increased risk of cardiovascular risk and gastrointestinal bleeding which limit their use in CRC chemoprevention (140). However certain case control studies such as the one based on Danish population analyzing non aspirin NSAID use (average daily dose & gt; or = 0.3) was associated with a substantial reduction in CRC risk. Aspirin and non-selective NSAIDs (SIR 0.74 [0.71-0.77]), but not COX-2i, were linked to lower risk of GI malignancies including CRC, according to a Swedish population-based analysis of persons taking frequent NSAIDs (cumulative exposure of 6 months) (141). Another prospective cohort study analysis found that using non-aspirin NSAIDs was linked to a decreased risk of CRC in postmenopausal women (142).

Chemoprevention necessitates the continuous administration of NSAIDs. The case for prescribing a chemopreventive medication is more convincing when the patient’s CRC risk is higher, and the drug’s cumulative side effects are less severe. Traditional NSAIDs have adverse effects that worsen over time, particularly in older patients who take other drugs due to comorbidities that interact with the chemopreventive agent (107, 143). As a result, the CRC risk must be significantly more than the 5% likelihood that a person at average risk will develop CRC in order to sustain the lifetime treatment of a typical NSAID. The use of NSAIDs for cancer chemoprevention is not advised despite the substantial evidence of activity because of the risk of serious renal, gastrointestinal, and cardiovascular adverse effects that arise from COX inhibition and the suppression of physiologically significant prostaglandin (111, 133). The chemopreventive efficacy of NSAIDs is also insufficient, albeit it is unclear whether this deficiency is brought on by dosage restrictions or resistance mechanisms. Hence preventing NSAIDS from getting into more clinical trials and FDA approval in CRC chemoprevention.

## 7. Advantages, challenges and future perspective of NSAIDs

### 7.1. Advantages of NSAIDs

Patients who smoked heavily and had a high BMI had decreased ability to benefit from the chemo-preventive effects of NSAIDs, especially aspirin. Ibuprofen use was linked to a lower incidence of CRC in a different cohort study of patients with germline mismatch repair gene mutations (144, 145). The use of both aspirin and non-aspirin NSAIDs was associated with a reduced risk of cancer, including CRC. The FDA has authorized the use of NSAIDs as analgesics, antipyretics, and anti-inflammatory drugs. These qualities allow NSAIDs to be used to treat a wide range of illnesses, such as migraines, pyrexia, gout, arthritic disorders, muscle pain, and dysmenorrhea, and as an opioid alternative in some cases of severe trauma (145). Colorectal cancers with PIK3CA mutations or COX2 overexpression appear to have a stronger correlation between NSAID and aspirin usage and decreased mortality. Thus lending credence to the idea that NSAIDs might be used as adjuvant therapy for CRC. Optimizing the timing of NSAID use as an adjuvant treatment is clinically important. The synergistic anticancer effect of aspirin, biologically, might be explained by the stimulation of apoptosis through a COX-dependent or COX-independent mechanism (107, 146), the decrease of metastatic risk by preventing the contact between platelets and circulating cancer cells (109, 111, 147, 148), or the modification of the antitumor immune response (112, 149). In the end, NSAIDs may have more than one target and most likely has several adjunctive effects.

### 7.2. Challenges In The Use of NSAIDs As CRC chemopreventive

Despite the immense potential of NSAIDs as chemo-preventive agents, their use in CRC chemoprevention encounters many challenges. The poor acceptability and cost of screening colonoscopies are the two factors that make chemoprevention of colorectal cancer (CRC) a viable approach. The most promising treatment agents are those NSAIDs, which are presently not advised for the prevention of CRC (150). NSAIDs’ limited chemo-preventive effectiveness is exacerbated by their considerable toxicity, which can be cumulative. These limitations can be curbed by the use of drug combinations, and the development of certain classes of NSAIDs that are chemically modified (for example – phospho-NSAIDs, nitro-NSAIDs, sulindac) and thus have prolonged safety than any other type of NSAIDs like those of conventional ones (150). One of the major challenges for using NSAIDs as a chemo-preventive drug is identifying the subjects who will gain the most from the chemo-preventive medication and those who are at potentially higher risk (151). The development of biomarkers that are predictive and techniques to reliably evaluate risk would be immensely beneficial in this case. Another challenge is optimizing chemo-preventive drug delivery time, dosage, and duration. According to research, very brief durations of agent administration may be necessary and can prevent colon carcinogenesis at an extremely early stage (152). The dosage and duration of NSAID administration might therefore be adjusted to ensure that the least amount of NSAID is utilized for the shortest duration of time. Furthermore, for individuals at risk, starting such an intervention at an early age may be beneficial. Also, the use of other new or combined agents or those agents that prevent other diseases in addition to colorectal cancer has its own merits (153). A meta-analysis of aspirin’s role in preventing CRC and other malignancies in recent years published in May 2009 showed frequent aspirin use is linked to a lower risk of cancer. However, this theory raises various issues, such as the best aspirin dose and the prevention of gastrointestinal bleeding brought on by prolonged aspirin usage. Thus, there is still much debate about the use of aspirin in the prevention and treatment of cancer (154).

NSAIDs are associated with other serious non-cancerous conditions also. As it was recently revealed that using NSAIDs increases the risk of myocardial infarction (155). Among the medications examined were Celecoxib, ibuprofen, diclofenac, naproxen, and rofecoxib (156). According to a study, many NSAID users reported gastrointestinal side effects ranging from nausea, slight pain, and dyspeptic symptoms to serious problems like bleeding, peptic ulcer rupture, and intestinal blockage (157). Peptic ulcer illness in the past, age, and concurrent aspirin usage are all significant risk factors for developing GI side effects in NSAID users (158, 159). NSAIDs are known for having substantial renal side effects, which in extreme situations might result in renal failure, in addition to cardiovascular and gastrointestinal problems (160). A higher risk has been noted in previous research for acute renal failure. Thus, the use of NSAIDs in the treatment and prevention of cancer must be carefully evaluated and there must also be a balance between the risks and the benefits (5).

### 7.3. Future perspectives

The number of studies on CRC chemoprevention has grown. Although NSAIDs have shown the most promise, only those with a greater risk of CRC predisposition syndromes, such as Lynch syndrome or FAP, have been advised to take them as chemopreventive medicines (161). The ideal CRC chemoprevention drug is elusive for the majority of patients. Finding new colonic neoplastic pathways that can be targeted as well as developing drug combinations that maximize efficacy and reduce toxicity are obstacles to CRC chemoprevention. It’s crucial to establish if more typical intermediate endpoints, like ACF or adenomas, may be employed given the generally low incidence of CRC in populations at average risk. Identifying subgroups based on genetic characteristics that influence treatment response, a history of polyps, and the subtype of a polyp is vital to determine which subgroups are most likely to benefit from chemoprevention drugs with the lowest degree of risk. CRC chemoprevention research must overcome obstacles including the necessity for funds to finance lengthy trials that enlist lots of participants and the requirement to validate results in various ethnic groups and geographical regions. Since many possible chemoprevention medicines are sold as over-the-counter drugs or dietary supplements, it is crucial to get reliable data on risk since their widespread usage might skew study results. It seems doubtful that CRC screening will ever be replaced as the main form of prevention by chemoprevention. The ability to prove the effectiveness of chemoprevention techniques in clinical trials will become more challenging as screening rates rise and CRC incidence and death decline (162, 163). Therefore, studies in groups who regularly receive CRC screening will need to show a stronger protective impact to significantly support chemoprevention in addition to screening. In conclusion, a chemopreventive drug that is generally effective, safe, affordable, accessible, and simple to use is appropriate for CRC. The promise of lowering CRC risk and lowering its morbidity and mortality makes CRC chemoprevention an activity worth continuing to pursue, even if it is difficult to discover a chemoprevention medication that complies with these requirements.

## 8. Conclusion

CRC being the second leading cause of cancer death globally is a major concern among the WHO (World Health Organization). Its preventive measures and treatments have become one of the challenging issues in the public health sector. CRC has been regarded as a sporadic and hereditary disease caused due to accumulation of genetic and epigenetic abnormalities in epithelial cells of the large intestine. It comprises of several modifiable (diet, alcohol, obesity, exercise) and non-modifiable risk factors such as age, genes, family history, etc. Several biomarkers such as KRAS, a major CRC biomarker, help in the early detection of colorectal cancer in patients. With the advent of technology and biological, physiological, and statistical constraints of endogenous biomarkers unavoidable need for the development of synthetic biomarkers in cancer sectors became a priority. Hence, several vector-based, mammalian cell-based, and bacterial cell-based synthetic biomarkers have been employed for the early detection of CRC on the basis of their advantages. Furthermore, among the various invasive techniques, colonoscopy is the most preferred method for early detection of CRC due to its better sensitivity-95% and specificity- 98% whereas sigmoidoscopy is more cost-efficient as compared to the expensive colonoscopic procedure. However, due to better specificity, colonoscopy is the most preferred procedure followed by sigmoidoscopy. Apart from this several non-invasive analytical methods based on DNA–RNA, protein, and metabolites found in a patient’s breath, blood, urine, and stool can be detected by utilizing genomic and mutation analytical techniques.

Chemoprevention techniques may help to further lower the incidence and mortality of CRC. Chemoprevention medications can be used for both low- and high-risk populations, as well as to stop colorectal cancer from returning following treatment. Aspirin, non-aspirin non-steroidal anti-inflammatory medications, statins, medicines that target metabolic pathways, vitamins, and minerals are examples of CRC chemoprevention treatments that have been explored (164).

NSAIDs are powerful anti-inflammatory, antipyretic and analgesic drugs having a chemopreventive impact on gastrointestinal malignancies, especially CRC, whereas long-term use of NSAIDs has also been linked to renal illness, myocardial infarction, gastrointestinal illness etc. Several NSAIDs, especially aspirin lower the risk and death from several malignancies, which is significant evidence that connects inflammation and cancer. The primary anticancer action of NSAIDs is assumed to be a COX-2 inhibition-mediated suppression of prostaglandin E2 production, which reduces tumor cell proliferation, and angiogenesis, and enhances apoptosis. Numerous studies have been conducted on the relationship between the expression of COX and colorectal cancer potential impact of NSAIDs-chemo-preventive drugs. It has been noted that statins and NSAIDs together show the synergistic effect as anticarcinogenic drugs in several *in vitro* and *in vivo* preclinical investigations, and this has drawn significant interest in examining their potential collaborative impact in cancer chemoprevention and combating the problems associated with the use of NSAIDs. This synergistic effect of combinational use of drugs proves to be beneficial in terms of reduced dosage and duration which is a potential technique for improving cancer prevention effectiveness.

## Author contributions

GR and NAK played a role in designing the study as well as drafted the review paper. NAK, DE, AR, Tanzeelah, HM, HA, AR, and MSU did the writing part. AB, MAK, and WH helped in the revision. All authors contributed to the article and approved the submitted version.

## Conflict of interest

The authors declare that the research was conducted in the absence of any commercial or financial relationships that could be construed as a potential conflict of interest.

## Publisher’s note

All claims expressed in this article are solely those of the authors and do not necessarily represent those of their affiliated organizations, or those of the publisher, the editors and the reviewers. Any product that may be evaluated in this article, or claim that may be made by its manufacturer, is not guaranteed or endorsed by the publisher.

## References

[1] Duarte Mendes A Vicente R Vitorino M Silva M Alpuim Costa D. Modulation of tumor environment in colorectal cancer – could gut microbiota be a key player? Frontiers in Gastroenterology. (2022) 1:23. doi: 10.3389/FGSTR.2022.1021050 PMC12952323 41822077

[2] Lundqvist E Myrberg IH Boman SE Saraste D Weibull CE Landerholm K. Autoimmune and metabolic diseases and the risk of early-onset colorectal cancer, a Nationwide nested case–control study. Cancer. (2023) 15:688. doi: 10.3390/cancers15030688 PMC9913656 36765646

[3] Knowlton CA Mackay MK Speer TW Vera RB Arthur DW Wazer DE. Cancer colon. Encyclo Radiat Oncol. (2013):77–7. doi: 10.1007/978-3-540-85516-3_1047/COVER

[4] Bien SA Su YR Conti DV Harrison TA Qu C Guo X. Genetic variant predictors of gene expression provide new insight into risk of colorectal cancer. Hum Genet. (2019) 138:307–26. doi: 10.1007/s00439-019-02030-8, PMID: 30820706 PMC6483948

[5] Alvarez-Gonzalez MA Pantaleon MA Flores-Le Roux JA Zaffalon D Amorós J Bessa X. Randomized clinical trial: a normocaloric low-fiber diet the day before colonoscopy is the most effective approach to bowel preparation in colorectal cancer screening colonoscopy. Dis Colon Rectum. (2019) 62:491–7. doi: 10.1097/DCR.0000000000001305 30844973 PMC6445600

[6] Aronson JK Ferner RE. Biomarkers—A General Review. Curr Protoc Pharmacol. (2017) 76:9.23.1–9.23.17. doi: 10.1002/cpph.19, PMID: 28306150

[7] Businello G Parente P Mastracci L Pennelli G Traverso G Milione M. The pathologic and molecular landscape of esophageal squamous cell carcinogenesis. Cancer. (2020) 12:2160. doi: 10.3390/CANCERS12082160, PMID: 32759723 PMC7465394

[8] Ogunwobi OO Mahmood F Akingboye A. Biomarkers in colorectal cancer: current research and future prospects. Int J Mol Sci. (2020) 21:5311. doi: 10.3390/ijms21155311, PMID: 32726923 PMC7432436

[9] Shaukat A Levin TR. Current and future colorectal cancer screening strategies. Nat Rev Gastroenterol Hepatol. (2022) 19:521–31. doi: 10.1038/s41575-022-00612-y, PMID: 35505243 PMC9063618

[10] Shimozaki K Hirata K Horie S Chida A Tsugaru K Hayashi Y. The entire intestinal tract surveillance using capsule endoscopy after immune checkpoint inhibitor administration: a prospective observational study. Diagnostics. (2021) 11:543. doi: 10.3390/diagnostics11030543, PMID: 33803735 PMC8003297

[11] Liang PS Dominitz JA. Colorectal cancer screening: is colonoscopy the best option? Med Clin North Am. (2019) 103:111–23. doi: 10.1016/j.mcna.2018.08.010, PMID: 30466668

[12] Iannone A Losurdo G Pricci M Girardi B Massaro A Principi M. Stool investigations for colorectal cancer screening: from occult blood test to DNA analysis. J Gastrointest Cancer. (2016) 47:143–51. doi: 10.1007/s12029-016-9810-z, PMID: 26922358

[13] Hoffman RM Levy BT Allison JE. Rising use of multitarget stool DNA testing for colorectal cancer. JAMA Netw Open. (2021) 4:e2122328–8. doi: 10.1001/jamanetworkopen.2021.22328, PMID: 34473264

[14] Donegan C Hughes AE Lee SJC. Colorectal cancer incidence, inequalities, and prevention priorities in urban Texas: surveillance study with the “surveil” software package. JMIR Public Health Surveill. (2022) 8:e34589. doi: 10.2196/34589, PMID: 35972778 PMC9428771

[15] Seijo LM Peled N Ajona D Boeri M Field JK Sozzi G. Biomarkers in lung cancer screening: achievements, promises, and challenges. J Thorac Oncol. (2019) 14:343–57. doi: 10.1016/j.jtho.2018.11.023, PMID: 30529598 PMC6494979

[16] Wang YC Tian ZB Tang XQ. Bioinformatics screening of biomarkers related to liver cancer. BMC Bioinform. (2021) 22:1–11. doi: 10.1186/S12859-021-04411-1/TABLES/2 PMC8543826 34696748

[17] Matsuoka T Yashiro M. Biomarkers of gastric cancer: current topics and future perspective. World J Gastroenterol. (2018) 24:2818–32. doi: 10.3748/WJG.V24.I26.2818 30018477 PMC6048430

[18] Lech G Słotwiński R Słodkowski M Krasnodębski IW. Colorectal cancer tumour markers and biomarkers: recent therapeutic advances. World J Gastroenterol. (2016) 22:1745–55. doi: 10.3748/wjg.v22.i5.1745, PMID: 26855534 PMC4724606

[19] Giampaolino P Foreste V Della Corte L di Filippo C Iorio G Bifulco G. Role of biomarkers for early detection of ovarian cancer recurrence. Gland Surg. (2020) 9:1102–11. doi: 10.21037/gs-20-544, PMID: 32953625 PMC7475347

[20] Filella X Fernández-Galán E Fernández Bonifacio R Foj L. Emerging biomarkers in the diagnosis of prostate cancer. Pharmgenomics Pers Med. (2018) Volume 11:83–94. doi: 10.2147/PGPM.S136026, PMID: 29844697 PMC5961643

[21] Sukumar J Gast K Quiroga D Lustberg M Williams N. Triple-negative breast cancer: promising prognostic biomarkers currently in development. Expert Rev Anticancer Ther. (2021) 21:135–48. doi: 10.1080/14737140.2021.1840984, PMID: 33198517 PMC8174647

[22] Li WC Lee PL Chou IC Chang WJ Lin SC Chang KW. Molecular and cellular cues of diet-associated oral carcinogenesis--with an emphasis on areca-nut-induced oral cancer development. J Oral Pathol Med. (2015) 44:167–77. doi: 10.1111/jop.12171, PMID: 24527773

[23] Eng VA David SP Li S Ally MS Stefanick M Tang JY. The association between cigarette smoking, cancer screening, and cancer stage: a prospective study of the women’s health initiative observational cohort. BMJ Open. (2020) 10:e037945. doi: 10.1136/bmjopen-2020-037945, PMID: 32796021 PMC7430331

[24] Jemal A Ward EM Johnson CJ Cronin KA Ma J Ryerson AB. Annual report to the nation on the status of cancer, 1975-2014, featuring survival. J Natl Cancer Inst. (2017) 109:djx030. doi: 10.1093/JNCI/DJX030 28376154 PMC5409140

[25] Cronin KA Lake AJ Scott S Sherman RL Noone AM Howlader N. Annual report to the nation on the status of cancer, part I: national cancer statistics. Cancer. (2018) 124:2785–800. doi: 10.1002/cncr.31551, PMID: 29786848 PMC6033186

[26] Meyer B Are C. Current status and future directions in colorectal cancer. Indian J Surg Oncol. (2018) 9:440. doi: 10.1007/s13193-017-0711-9, PMID: 30538369 PMC6265201

[27] Botteri E Borroni E Sloan EK Bagnardi V Bosetti C Peveri G. Smoking and colorectal cancer risk, overall and by molecular subtypes: a meta-analysis. Am J Gastroenterol. (2020) 115:1940–9. doi: 10.14309/ajg.0000000000000803, PMID: 32773458

[28] Figueiredo JC Crockett SD Snover DC Morris CB McKeown-Eyssen G Sandler RS. Smoking-associated risks of conventional adenomas and serrated polyps in the colorectum. Cancer Causes Control. (2015) 26:377–86. doi: 10.1007/s10552-014-0513-0, PMID: 25537738 PMC4331601

[29] Olsen M Ghannad M Lok C Bossuyt PM. Shortcomings in the evaluation of biomarkers in ovarian cancer: a systematic review. Clin Chem Lab Med. (2019) 58:3–10. doi: 10.1515/cclm-2019-0038, PMID: 30956227

[30] Kuchenbaecker KB Hopper JL Barnes DR Phillips K-A Mooij TM Roos-Blom M-J. Risks of breast, ovarian, and contralateral breast cancer for BRCA1 and BRCA2 mutation carriers. JAMA. (2017) 317:2402–16. doi: 10.1001/JAMA.2017.7112 28632866

[31] Fenton JJ Weyrich MS Durbin S Liu Y Bang H Melnikow J. Prostate-specific antigen-based screening for prostate cancer: evidence report and systematic review for the US preventive services task force. JAMA. (2018) 319:1914–31. doi: 10.1001/JAMA.2018.3712 29801018

[32] Zargar A Miroliaee A Ahmadi Gooraji S Hajaghamohammadi A. Determination of effective factors on survival of GI cancers: results of five years follow up in Iranian population. Global J Health Sci. (2016) 8:256–66. doi: 10.5539/GJHS.V8N6P256, PMID: 26755479 PMC4954912

[33] Allegra CJ Rumble RB Hamilton SR Mangu PB Roach N Hantel A. Extended RAS gene mutation testing in metastatic colorectal carcinoma to predict response to anti-epidermal growth factor receptor monoclonal antibody therapy: American Society of Clinical Oncology provisional clinical opinion update 2015. J Clin Oncol. (2016) 34:179–85. doi: 10.1200/JCO.2015.63.9674, PMID: 26438111

[34] Locker GY Hamilton S Harris J Jessup JM Kemeny N Macdonald JS. ASCO 2006 update of recommendations for the use of tumor markers in gastrointestinal cancer. J Clin Oncol. (2006) 24:5313–27. doi: 10.1200/JCO.2006.08.2644, PMID: 17060676

[35] Lim E Palmieri C Tilley WD. Renewed interest in the progesterone receptor in breast cancer. Br J Cancer. (2016) 115:909–11. doi: 10.1038/bjc.2016.303, PMID: 27657336 PMC5061913

[36] Bang YJ van Cutsem E Feyereislova A Chung HC Shen L Sawaki A. Trastuzumab in combination with chemotherapy versus chemotherapy alone for treatment of HER2-positive advanced gastric or gastro-oesophageal junction cancer (ToGA): a phase 3, open-label, randomised controlled trial. Lancet. (2010) 376:687–97. doi: 10.1016/S0140-6736(10)61121-X, PMID: 20728210

[37] Acharya A Markar SR Matar M Ni M Hanna GB. Use of tumor markers in gastrointestinal cancers: surgeon perceptions and cost-benefit trade-off analysis. Ann Surg Oncol. (2017) 24:1165–73. doi: 10.1245/s10434-016-5717-y, PMID: 28008574 PMC5374165

[38] Pedrazzoli P Rosti G Soresini E Ciani S Secondino S. Serum tumour markers in germ cell tumours: from diagnosis to cure. Crit Rev Oncol Hematol. (2021) 159:103224. doi: 10.1016/j.critrevonc.2021.103224, PMID: 33493632

[39] Kabel AM. Tumor markers of breast cancer: new prospectives. J Oncol Sci. (2017) 3:5–11. doi: 10.1016/j.jons.2017.01.001, PMID: 36510632

[40] Kwong GA Ghosh S Gamboa L Patriotis C Srivastava S Bhatia SN. Synthetic biomarkers: a twenty-first century path to early cancer detection. Nat Rev Cancer. (2021) 21:655–68. doi: 10.1038/s41568-021-00389-3, PMID: 34489588 PMC8791024

[41] Zhang XJ Hu LY Hu Y Yang XT Tang YY Tang YY. Tumor-penetrating hierarchically structured Nanomarker for imaging-guided urinary monitoring of cancer. ACS Sens. (2020) 5:1567–72. doi: 10.1021/acssensors.9b02194, PMID: 32456420

[42] Lopez-Giacoman S Madero M. Biomarkers in chronic kidney disease, from kidney function to kidney damage. World J Nephrol. (2015) 4:57–73. doi: 10.5527/wjn.v4.i1.57, PMID: 25664247 PMC4317628

[43] Kobayashi K Kawaguchi Y Kobayashi Y Matsumura M Ishizawa T Akamatsu N. Identification of liver lesions using fluorescence imaging: comparison of methods for administering indocyanine green. HPB. (2021) 23:262–9. doi: 10.1016/j.hpb.2020.06.006, PMID: 32675045

[44] Khan NA Hussain M Rahman AU Farooqui WA Rasheed A Memon AS. Dietary practices, addictive behavior and bowel habits and risk of early onset colorectal cancer: a case control study. Asian Pac J Cancer Prev. (2015) 16:7967–73. doi: 10.7314/APJCP.2015.16.17.7967, PMID: 26625827

[45] Eibl RH Schneemann M. Cell-free DNA as a biomarker in cancer. Extracell Vesicles Circ Nucl Acids. (2022) 3:178–98. doi: 10.20517/evcna.2022.20, PMID: 36755121

[46] Li H Zhang H Lu G Li Q Gu J Song Y. Mechanism analysis of colorectal cancer according to the microRNA expression profile. Oncol Lett. (2016) 12:2329. doi: 10.3892/ol.2016.5027, PMID: 27698796 PMC5038387

[47] Song L Li Y. SEPT9: a specific circulating biomarker for colorectal cancer. Adv Clin Chem. (2015) 72:171–204. doi: 10.1016/BS.ACC.2015.07.004 26471083

[48] Dong L Lin W Qi P Xu MD Wu X Ni S. Circulating long RNAs in serum extracellular vesicles: their characterization and potential application as biomarkers for diagnosis of colorectal cancer. Cancer Epidemiol Biomark Prev. (2016) 25:1158–66. doi: 10.1158/1055-9965.EPI-16-0006, PMID: 27197301

[49] Wang Y Li Z Li W Liu S Han B. Methylation of promoter region of CDX2 gene in colorectal cancer. Oncol Lett. (2016) 12:3229. doi: 10.3892/ol.2016.5109, PMID: 27899987 PMC5103925

[50] Mezheyeuski A Ponten F Edqvist PH Sundström M Thunberg U Qvortrup C. Metastatic colorectal carcinomas with high SATB2 expression are associated with better prognosis and response to chemotherapy: a population-based Scandinavian study. Acta Oncol. (2020) 59:284–90. doi: 10.1080/0284186X.2019.1691258, PMID: 31769323

[51] Issa IA NouredDine M. Colorectal cancer screening: An updated review of the available options. World J Gastroenterol. (2017) 23:5086. doi: 10.3748/wjg.v23.i28.5086, PMID: 28811705 PMC5537177

[52] Dettling DE Kwok E Quach L Datt A Degenhardt JD Panchal A. Regression of EGFR positive established solid tumors in mice with the conditionally active T cell engager TAK-186. J Immunother Cancer. (2022) 10:e004336. doi: 10.1136/jitc-2021-004336, PMID: 35728872 PMC9214390

[53] Lopes N Bergsland C Bruun J Bjørnslett M Vieira AF Mesquita P. A panel of intestinal differentiation markers (CDX2, GPA33, and LI-cadherin) identifies gastric cancer patients with favourable prognosis. Gastric Cancer. (2020) 23:811–23. doi: 10.1007/s10120-020-01064-6, PMID: 32215766

[54] Wong K-F Liu AM Hong W Xu Z Luk JM Wong K-F. Integrin α2β1 inhibits MST1 kinase phosphorylation and activates yes-associated protein oncogenic signaling in hepatocellular carcinoma. Oncotarget. (2016) 7:77683–95. doi: 10.18632/oncotarget.12760, PMID: 27765911 PMC5363613

[55] Jia H Wang Z. Telomere length as a prognostic factor for overall survival in colorectal cancer patients. Cell Physiol Biochem. (2016) 38:122–8. doi: 10.1159/000438614, PMID: 26741140

[56] Hultcrantz R. Aspects of colorectal cancer screening, methods, age and gender. J Intern Med. (2021) 289:493–507. doi: 10.1111/joim.13171, PMID: 32929813 PMC8048936

[57] Kim S Wong P Coulombe PA. A keratin cytoskeletal protein regulates protein synthesis and epithelial cell growth. Nature. (2006) 441:362–5. doi: 10.1038/nature04659, PMID: 16710422

[58] Sheng J Sun H Yu F-B Li B Zhang Y Zhu Y-T. The role of Cyclooxygenase-2 in colorectal cancer. Int J Med Sci. (2020) 2020:1095–101. doi: 10.7150/ijms.44439 PMC7211146 32410839

[59] Rao X Wang J Song HM Deng B Li JG. KRT15 overexpression predicts poor prognosis in colorectal cancer. Neoplasma. (2020) 67:410–4. doi: 10.4149/neo_2019_190531N475, PMID: 31884802

[60] Dawson H Lugli A. Molecular and pathogenetic aspects of tumor budding in colorectal cancer. Front Med. (2015) 2:11. doi: 10.3389/fmed.2015.00011, PMID: 25806371 PMC4354406

[61] Li J Hao Q Cao W Vadgama J Wu Y. Celecoxib in breast cancer prevention and therapy. Cancer Manag Res. (2018) 10:4653–67. doi: 10.2147/CMAR.S178567 30464589 PMC6208493

[62] Binefa G Rodríguez-Moranta F Teule À Medina-Hayas M. Colorectal cancer: from prevention to personalized medicine. World J Gastroenterol. (2014) 20:6786–808. doi: 10.3748/wjg.v20.i22.6786, PMID: 24944469 PMC4051918

[63] Ma L Qin G Gai F Jiang Y Huang Z Yang H. A novel method for early detection of colorectal cancer based on detection of methylation of two fragments of syndecan-2 (SDC2) in stool DNA. BMC Gastroenterol. (2022) 22:1–10. doi: 10.1186/S12876-022-02264-3/TABLES/5 35436855 PMC9014784

[64] Oh T Kim N Moon Y Kim MS Hoehn BD Park CH. Genome-wide identification and validation of a novel methylation biomarker, SDC2, for blood-based detection of colorectal cancer. J Mol Diagn. (2013) 15:498–507. doi: 10.1016/j.jmoldx.2013.03.004, PMID: 23747112

[65] Tuohy TMF Rowe KG Mineau GP Pimentel R Burt RW Samadder NJ. Risk of colorectal cancer and adenomas in the families of patients with adenomas: a population-based study in Utah. Cancer. (2014) 120:35–42. doi: 10.1002/cncr.28227, PMID: 24150925

[66] Itatani Y Kawada K Sakai Y. Treatment of elderly patients with colorectal cancer. Biomed Res Int. (2018) 2018:2176056. doi: 10.1155/2018/2176056, PMID: 29713641 PMC5866880

[67] Schottinger, JE, Jensen, CD, Ghai, NR, Chubak J, Lee, JK, Kamineni, A et al. Association of physician adenoma detection rates with postcolonoscopy colorectal cancer. Jama. (2022) 327:2114–22. 35670788 10.1001/jama.2022.6644 PMC9175074

[68] Corley DA Jensen CD Marks AR Zhao WK Lee JK Doubeni CA. Adenoma detection rate and risk of colorectal cancer and death. N Engl J Med. (2014) 370:1298–306. doi: 10.1056/NEJMoa1309086, PMID: 24693890 PMC4036494

[69] Kaminski MF Regula J Kraszewska E Polkowski M Wojciechowska U Didkowska J. Quality indicators for colonoscopy and the risk of interval cancer. N Engl J Med. (2010) 362:1795–803. doi: 10.1056/NEJMoa0907667, PMID: 20463339

[70] Carter, JV, Roberts, HL, Pan, J, Rice, JD, Burton, JF, Galbraith, NJ, et al. A highly predictive model for diagnosis of colorectal neoplasms using plasma microRNA: improving specificity and sensitivity. Annals of Surgery. (2016) 264:575. 27471839 10.1097/SLA.0000000000001873 PMC5115272

[71] Gupta S. Screening for colorectal cancer. Hematol Oncol Clin North Am. (2022) 36:393–414. doi: 10.1016/j.hoc.2022.02.001, PMID: 35501176 PMC9167799

[72] Imperiale TF Ransohoff DF Itzkowitz SH Levin TR Lavin P Lidgard GP. Multitarget stool DNA testing for colorectal-cancer screening. N Engl J Med. (2014) 370:1287–97. doi: 10.1056/NEJMoa1311194, PMID: 24645800

[73] Bromer MQ Weinberg DS. Screening for colorectal cancer - now and the near future. Semin Oncol. (2005) 32:3–10. doi: 10.1053/j.seminoncol.2004.09.031, PMID: 15726501

[74] Maida M Macaluso FS Ianiro G Mangiola F Sinagra E Hold G. Screening of colorectal cancer: present and future. Expert Rev Anticancer Ther. (2017) 17:1131–46. doi: 10.1080/14737140.2017.1392243, PMID: 29022408

[75] Hassan C Antonelli G Dumonceau JM Regula J Bretthauer M Chaussade S. Post-polypectomy colonoscopy surveillance: European Society of Gastrointestinal Endoscopy (ESGE) guideline - update 2020. Endoscopy. (2020) 52:687–700. doi: 10.1055/a-1185-3109, PMID: 32572858

[76] Zygulska AL Pierzchalski P. Novel diagnostic biomarkers in colorectal cancer. Int J Mol Sci. (2022) 23:852. doi: 10.3390/ijms23020852, PMID: 35055034 PMC8776048

[77] Lin JS Perdue LA Henrikson NB Bean SI Blasi PR. Screening for colorectal cancer: updated evidence report and systematic review for the US preventive services task force. JAMA. (2021) 325:1978–98. doi: 10.1001/JAMA.2021.4417 34003220 PMC13509038

[78] Wang Y Chen PM Liu R bin. Advance in plasma SEPT9 gene methylation assay for colorectal cancer early detection. World J Gastrointest Oncol. (2018) 10:15. doi: 10.4251/wjgo.v10.i1.15, PMID: 29375744 PMC5767789

[79] Daniel, C. L. (2013). Predictors of colorectal cancer surveillance among survivors of childhood cancer at high risk for subsequent colorectal malignancies. The University of Alabama at Birmingham.

[80] Song L Jia J Peng X Xiao W Li Y. The performance of the SEPT9 gene methylation assay and a comparison with other CRC screening tests: a meta-analysis. Sci Rep. (2017) 7:1–12. doi: 10.1038/s41598-017-03321-8 28596563 PMC5465203

[81] Sawicki T Ruszkowska M Danielewicz A Niedźwiedzka E Arłukowicz T Przybyłowicz KE. A review of colorectal cancer in terms of epidemiology, risk factors, development, symptoms and diagnosis. Cancers. (2021) 13:2025. doi: 10.3390/cancers13092025, PMID: 33922197 PMC8122718

[82] Malik P. A novel multitarget stool DNA test for colorectal cancer screening. Postgrad Med. (2016) 128:268–72. doi: 10.1080/00325481.2016.1135035, PMID: 26753807

[83] Guinney J Dienstmann R Wang X de Reyniès A Schlicker A Soneson C. The consensus molecular subtypes of colorectal cancer. Nat Med. (2015) 21:1350–6. doi: 10.1038/nm.3967, PMID: 26457759 PMC4636487

[84] Alorda-Clara M Torrens-Mas M Morla-Barcelo PM Martinez-Bernabe T Sastre-Serra J Roca P. Use of omics technologies for the detection of colorectal cancer biomarkers. Cancers. (2022) 14:817. doi: 10.3390/cancers14030817, PMID: 35159084 PMC8834235

[85] Jin J. Nonsteroidal anti-inflammatory drugs. JAMA. (2015) 314:1084–4. doi: 10.1001/JAMA.2015.9936 26348765

[86] Piazuelo E Lanas A. NSAIDS and gastrointestinal cancer. Prostaglandins Other Lipid Mediat. (2015) 120:91–6. doi: 10.1016/j.prostaglandins.2015.06.001, PMID: 26093284

[87] Iavecchia L Cereza García G Sabaté Gallego M Vidal Guitart X Ramos Terrades N de la Torre J. Drug-related acute renal failure in hospitalised patients. Nefrología. (2015) 35:523–32. doi: 10.1016/j.nefroe.2016.01.001, PMID: 26474529

[88] Sheng, J, Sun, H, Yu, FB, Li, B, Zhang, Y, Zhu, YT. The role of cyclooxygenase-2 in colorectal cancer. Int.l J. Med. Sci. (2020) 17:1095. 10.7150/ijms.44439 PMC7211146 32410839

[89] Rouzer CA Marnett LJ. Cyclooxygenases: structural and functional insights. J Lipid Res. (2009) 50:S29–34. doi: 10.1194/jlr.R800042-JLR200, PMID: 18952571 PMC2674713

[90] Bäumler P Zhang W Stübinger T Irnich D. Acupuncture-related adverse events: systematic review and meta-analyses of prospective clinical studies. BMJ Open. (2021) 11:e045961. doi: 10.1136/bmjopen-2020-045961, PMID: 34489268 PMC8422480

[91] McEvoy L Carr DF Pirmohamed M. Pharmacogenomics of NSAID-induced upper gastrointestinal toxicity. Front Pharmacol. (2021) 12:684162. 34234675 10.3389/fphar.2021.684162 PMC8256335

[92] Zhang Y Pan KF Zhang L Ma JL Zhou T Li J. Helicobacter pylori, cyclooxygenase-2 and evolution of gastric lesions: results from an intervention trial in China. Carcinogenesis. (2015) 36:1572–9. doi: 10.1093/carcin/bgv147, PMID: 26449252

[93] Kolawole OR Kashfi K. NSAIDs and cancer resolution: new paradigms beyond cyclooxygenase. Int J Mol Sci. (2022) 23:1432. doi: 10.3390/ijms23031432, PMID: 35163356 PMC8836048

[94] Medzhitov R. Origin and physiological roles of inflammation. Nature. (2008) 454:428–35. doi: 10.1038/nature07201, PMID: 18650913

[95] Kune GA Kune S Watson LF. Colorectal cancer risk, chronic illnesses, operations and medications: case–control results from the Melbourne colorectal cancer study. Int J Epidemiol. (2007) 36:951–7. doi: 10.1093/ije/dym193, PMID: 17921195

[96] Ohara K Takahara M Kumai T Yamashina M Kishibe K Katada A Hayashi T. Treatment outcomes of alternating chemoradiotherapy for nasopharyngeal carcinoma: a single-center safety and efficacy study. Brazilian J Otorhinolaryngol (2022) doi: 10.1016/j.bjorl.2022.12.004, PMID: [Epub ahead of print]. 36682990 PMC10164767

[97] Siegfried G Descarpentrie J Evrard S Khatib AM. Proprotein convertases: key players in inflammation-related malignancies and metastasis. Cancer Lett. (2020) 473:50–61. doi: 10.1016/j.canlet.2019.12.027, PMID: 31899298 PMC7115805

[98] Hefetz-Sela S Stein I Pikarsky E. Restoring inflammatory balance as a potential preventive strategy for inflammation induced cancer. Oncoimmunology. (2015) 4:e1039764. doi: 10.1080/2162402X.2015.1039764 26451313 PMC4589047

[99] Sankaranarayanan R Kumar DR Altinoz MA Bhat GJ. Mechanisms of colorectal cancer prevention by aspirin-a literature review and perspective on the role of COX-dependent and -independent pathways. Int J Mol Sci. (2020) 21:1–18. doi: 10.3390/IJMS21239018 PMC7729916 33260951

[100] Chen J Stark LA. Aspirin prevention of colorectal cancer: focus on NF-κB Signalling and the nucleolus. Biomedicine. (2017) 5:43. doi: 10.3390/biomedicines5030043, PMID: 28718829 PMC5618301

[101] Liu B Qu L Yan S. Cyclooxygenase-2 promotes tumor growth and suppresses tumor immunity. Cancer Cell Int. (2015) 15:1–6. doi: 10.1186/S12935-015-0260-7/TABLES/1 26549987 PMC4635545

[102] Pang LY Hurst EA Argyle DJ. Cyclooxygenase-2: a role in cancer stem cell survival and repopulation of cancer cells during therapy. Stem Cells Int. (2016) 2016:2048731. doi: 10.1155/2016/2048731, PMID: 27882058 PMC5108861

[103] Pannunzio A Coluccia M. Cyclooxygenase-1 (COX-1) and COX-1 inhibitors in cancer: a review of oncology and medicinal chemistry literature. Pharmaceuticals. (2018) 11:101. doi: 10.3390/ph11040101, PMID: 30314310 PMC6316056

[104] Bruno A Contursi A Tacconelli S Sacco A Hofling U Mucci M. The specific deletion of cyclooxygenase-1 in megakaryocytes/platelets reduces intestinal polyposis in ApcMin/+ mice. Pharmacol Res. (2022) 185:106506. doi: 10.1016/j.phrs.2022.106506, PMID: 36241001

[105] Rouzer CA Marnett LJ. Structural and chemical biology of the interaction of cyclooxygenase with substrates and non-steroidal anti-inflammatory drugs. Chem Rev. (2020) 120:7592–641. doi: 10.1021/acs.chemrev.0c00215, PMID: 32609495 PMC8253488

[106] Maniewska J Jeżewska D. Non-steroidal anti-inflammatory drugs in colorectal cancer chemoprevention. Cancer. (2021) 13:594. doi: 10.3390/cancers13040594, PMID: 33546238 PMC7913298

[107] Olkinuora AP Peltomäki PT Aaltonen LA Rajamäki K. From APC to the genetics of hereditary and familial colon cancer syndromes. Hum Mol Genet. (2021) 30:R206–24. doi: 10.1093/hmg/ddab208, PMID: 34329396 PMC8490010

[108] Cho M Gwak J Park S Won J Kim DE Sung SY. Diclofenac attenuates Wnt/β-catenin signaling in colon cancer cells by activation of NF-κB. FEBS Lett. (2005) 579:4213–8. doi: 10.1016/j.febslet.2005.06.049, PMID: 16051228

[109] PDQ Cancer Genetics Editorial Board Genetics of colorectal cancer (PDQ®): health professional version. PDQ Cancer Information Summaries. (2002). http://www.ncbi.nlm.nih.gov/pubmed/26389505 [Accessed January 22, 2023]

[110] Zaman FY Orchard SG Haydon A Zalcberg JR. Non-aspirin non-steroidal anti-inflammatory drugs in colorectal cancer: a review of clinical studies. Br J Cancer. (2022) 127:1735–43. doi: 10.1038/s41416-022-01882-8, PMID: 35764787 PMC9643522

[111] Marley AR Nan H. Epidemiology of colorectal cancer. Int J Mol Epidemiol Genet. (2016) 7:105. PMID: 27766137 PMC5069274

[112] Kanth P Grimmett J Champine M Burt R Samadder NJ. Hereditary colorectal polyposis and cancer syndromes: a primer on diagnosis and management. Am J Gastroenterol. (2017) 112:1509–25. doi: 10.1038/ajg.2017.212, PMID: 28786406

[113] Santoro A Bufo P Russo G Cagiano S Papagerakis S Bucci P. Expression and clinical implication of cyclooxygenase-2 and E-cadherin in oral squamous cell carcinomas. Cancer Biol Ther. (2020) 21:667–74. doi: 10.1080/15384047.2015.1071741 26218314 PMC7537792

[114] Jara-Gutiérrez Á Baladrón V. The role of prostaglandins in different types of cancer. Cells. (2021) 10:1487. doi: 10.3390/cells10061487, PMID: 34199169 PMC8231512

[115] Misron NA Looi LM Nik Mustapha NR. Cyclooxygenase-2 expression in invasive breast carcinomas of no special type and correlation with pathological profiles suggest a role in tumorigenesis rather than cancer progression. Asian Pac J Cancer Prev. (2015) 16:1553–8. doi: 10.7314/APJCP.2015.16.4.1553, PMID: 25743830

[116] Stiller CO Hjemdahl P. Lessons from 20 years with COX-2 inhibitors: importance of dose–response considerations and fair play in comparative trials. J Intern Med. (2022) 292:557–74. doi: 10.1111/joim.13505, PMID: 35585779

[117] Warner TD Giuliano F Vojnovic I Bukasa A Mitchell JA Vane JR. Nonsteroid drug selectivities for cyclo-oxygenase-1 rather than cyclo-oxygenase-2 are associated with human gastrointestinal toxicity: a full in vitro analysis. Proc Natl Acad Sci U S A. (1999) 96:7563–8. doi: 10.1073/pnas.96.13.7563, PMID: 10377455 PMC22126

[118] Hamoya T Fujii G Miyamoto S Takahashi M Totsuka Y Wakabayashi K. Effects of NSAIDs on the risk factors of colorectal cancer: a mini review. Genes Environ. (2016) 38:1–7. doi: 10.1186/S41021-016-0033-0/TABLES/2 27350826 PMC4918106

[119] Smith ML Hawcroft G Hull MA. The effect of non-steroidal anti-inflammatory drugs on human colorectal cancer cells: evidence of different mechanisms of action. Eur J Cancer. (2000) 36:664–74. doi: 10.1016/S0959-8049(99)00333-0, PMID: 10738133

[120] Faruk M Ibrahim S Aminu SM Adamu A Abdullahi A Suleiman AM. Prognostic significance of BIRC7/Livin, Bcl-2, p53, Annexin V, PD-L1, DARC, MSH2 and PMS2 in colorectal cancer treated with FOLFOX chemotherapy with or without aspirin. PLoS One. (2021) 16:e0245581. doi: 10.1371/journal.pone.0245581, PMID: 33465114 PMC7815153

[121] Song L Liu D Zhao Y He J Kang H Dai Z. Sinomenine inhibits breast cancer cell invasion and migration by suppressing NF-κB activation mediated by IL-4/miR-324-5p/CUEDC2 axis. Biochem Biophys Res Commun. (2015) 464:705–10. doi: 10.1016/j.bbrc.2015.07.004, PMID: 26166821

[122] Li X Gao L Cui Q Gary BD Dyess DL Taylor W. Sulindac inhibits tumor cell invasion by suppressing NF-κB-mediated transcription of microRNAs. Oncogene. (2012) 31:4979–86. doi: 10.1038/onc.2011.655, PMID: 22286762 PMC3372649

[123] Loveridge CJ MacDonald ADH Thoms HC Dunlop MG Stark LA. The proapoptotic effects of sulindac, sulindac sulfone and indomethacin are mediated by nucleolar translocation of the RelA(p65) subunit of NF-κB. Oncogene. (2008) 27:2648–55. doi: 10.1038/sj.onc.1210891, PMID: 18059344

[124] Bienz M Clevers H. Linking colorectal cancer to Wnt signaling. Cells. (2000) 103:311–20. doi: 10.1016/S0092-8674(00)00122-7, PMID: 11057903

[125] Piazza GA Ward A Chen X Maxuitenko Y Coley A Aboelella NS. PDE5 and PDE10 inhibition activates cGMP/PKG signaling to block Wnt/β-catenin transcription, cancer cell growth, and tumor immunity. Drug Discov Today. (2020) 25:1521–7. doi: 10.1016/j.drudis.2020.06.008, PMID: 32562844 PMC7484431

[126] Keum NN Giovannucci E. Global burden of colorectal cancer: emerging trends, risk factors and prevention strategies. Nat Rev Gastroenterol Hepatol. (2019) 16:713–32. doi: 10.1038/s41575-019-0189-8, PMID: 31455888

[127] Fajardo AM Piazza GA. Chemoprevention in gastrointestinal physiology and disease. Anti-inflammatory approaches for colorectal cancer chemoprevention. Am J Physiol Gastrointest Liver Physiol. (2015) 309:G59–70. doi: 10.1152/ajpgi.00101.2014, PMID: 26021807 PMC4504955

[128] Siegel RL Miller KD Jemal A. Cancer statistics, 2016. CA Cancer J Clin. (2016) 66:7–30. doi: 10.3322/caac.21332, PMID: 26742998

[129] Wesselink E van Baar H van Zutphen M Tibosch M Kouwenhoven EA Keulen ETP. Inflammation is a mediating factor in the association between lifestyle and fatigue in colorectal cancer patients. Cancers. (2020) 12:1–13. doi: 10.3390/CANCERS12123701 PMC7763620 33317113

[130] Wong KE Ngai SC Chan KG Lee LH Goh BH Chuah LH. Curcumin nanoformulations for colorectal cancer: a review. Front Pharmacol. (2019) 10:152. doi: 10.3389/FPHAR.2019.00152/BIBTEX 30890933 PMC6412150

[131] Chaudhary A Sutaria D Huang Y Wang J Prabhu S. Chemoprevention of colon cancer in a rat carcinogenesis model using a novel nanotechnology-based combined treatment system. Cancer Prev Res. (2011) 4:1655–64. doi: 10.1158/1940-6207.CAPR-11-0129, PMID: 21914855 PMC3188363

[132] Mohammed A Yarla NS Madka V Rao CV. Clinically relevant anti-inflammatory agents for chemoprevention of colorectal cancer: new perspectives. Int J Mol Sci. (2018) 19:2332. doi: 10.3390/IJMS19082332 30096840 PMC6121559

[133] Tsioulias GJ Go MF Rigas B. NSAIDs and colorectal cancer control: promise and challenges. Curr Pharmacol Rep. (2015) 1:295–301. doi: 10.1007/s40495-015-0042-x, PMID: 26688785 PMC4683110

[134] Blais L Desgagné A LeLorier J. 3-Hydroxy-3-methylglutaryl coenzyme a reductase inhibitors and the risk of cancer: a nested case-control study. Arch Intern Med. (2000) 160:2363–8. doi: 10.1001/archinte.160.15.2363, PMID: 10927735

[135] Jalving M Koornstra JJ de Jong S de Vries EGE Kleibeuker JH. Review article: the potential of combinational regimen with non-steroidal anti-inflammatory drugs in the chemoprevention of colorectal cancer. Aliment Pharmacol Ther. (2005) 21:321–39. doi: 10.1111/j.1365-2036.2005.02335.x, PMID: 15709983

[136] Reddy BS Chung XW Kong AN Tin OK Zheng X Steele VE. Prevention of azoxymethane-induced colon cancer by combination of low doses of atorvastatin, aspirin, and celecoxib in F 344 rats. Cancer Res. (2006) 66:4542–6. doi: 10.1158/0008-5472.CAN-05-4428, PMID: 16618783

[137] Mollinedo F Gajate C. Lipid rafts as major platforms for signaling regulation in cancer. Adv Biol Regul. (2015) 57:130–46. doi: 10.1016/j.jbior.2014.10.003, PMID: 25465296

[138] Zhuang L Kim J Adam RM Solomon KR Freeman MR. Cholesterol targeting alters lipid raft composition and cell survival in prostate cancer cells and xenografts. J Clin Invest. (2005) 115:959–68. doi: 10.1172/JCI200519935, PMID: 15776112 PMC1064980

[139] Sahai E Marshall CJ. RHO-GTPases and cancer. Nat Rev Cancer. (2002) 2:133–42. doi: 10.1038/nrc725, PMID: 12635176

[140] Niu S Ma X Zhang Y Liu YN Chen X Gong H. MicroRNA-19a and microRNA-19b promote the malignancy of clear cell renal cell carcinoma through targeting the tumor suppressor RhoB. PLoS One. (2018) 13:e0192790. doi: 10.1371/journal.pone.0192790, PMID: 29474434 PMC5825063

[141] Grancher A Michel P di Fiore F Sefrioui D. Colorectal cancer chemoprevention: is aspirin still in the game? Cancer Biol Ther. (2022) 23:446–61. doi: 10.1080/15384047.2022.2104561 35905195 PMC9341367

[142] Ishikawa H Mutoh M Suzuki S Tokudome S Saida Y Abe T. The preventive effects of low-dose enteric-coated aspirin tablets on the development of colorectal tumours in Asian patients: a randomised trial. Gut. (2014) 63:1755–9. doi: 10.1136/gutjnl-2013-305827, PMID: 24488498

[143] Gurpinar E Grizzle WE Piazza GA. NSAIDs inhibit tumorigenesis, but how? Clin Cancer Res. (2014) 20:1104. doi: 10.1158/1078-0432.CCR-13-1573, PMID: 24311630 PMC3947450

[144] Rothwell PM Cook NR Gaziano JM Price JF Belch JFF Roncaglioni MC. Effects of aspirin on risks of vascular events and cancer according to bodyweight and dose: analysis of individual patient data from randomised trials. Lancet. (2018) 392:387–99. doi: 10.1016/S0140-6736(18)31133-4, PMID: 30017552 PMC6083400

[145] Kemp Bohan PM Mankaney G Vreeland TJ Chick RC Hale DF Cindass JL. Chemoprevention in familial adenomatous polyposis: past, present and future. Familial Cancer. (2021) 20:23–33. doi: 10.1007/s10689-020-00189-y, PMID: 32507936 PMC7276278

[146] Langley RE Burdett S Tierney JF Cafferty F Parmar MKB Venning G. Aspirin and cancer: has aspirin been overlooked as an adjuvant therapy? Br J Cancer. (2011) 105:1107–13. doi: 10.1038/bjc.2011.289, PMID: 21847126 PMC3208483

[147] Gay LJ Felding-Habermann B. Contribution of platelets to tumour metastasis. Nat Rev Cancer. (2011) 11:123–34. doi: 10.1038/nrc3004, PMID: 21258396 PMC6894505

[148] Xu XR Yousef GM Ni H. Cancer and platelet crosstalk: opportunities and challenges for aspirin and other antiplatelet agents. Blood. (2018) 131:1777–89. doi: 10.1182/blood-2017-05-743187, PMID: 29519806

[149] Zelenay S van der Veen AG Böttcher JP Snelgrove KJ Rogers N Acton SE. Cyclooxygenase-dependent tumor growth through evasion of immunity. Cells. (2015) 162:1257–70. doi: 10.1016/j.cell.2015.08.015, PMID: 26343581 PMC4597191

[150] Thompson PA Ashbeck EL Roe DJ Fales L Buckmeier J Wang F. Selenium supplementation for prevention of colorectal adenomas and risk of associated type 2 diabetes. J Natl Cancer Inst. (2016) 108:djw152. doi: 10.1093/JNCI/DJW152 27530657 PMC6272807

[151] Umezawa S Higurashi T Komiya Y Arimoto J Horita N Kaneko T. Chemoprevention of colorectal cancer: past, present, and future. Cancer Sci. (2019) 110:3018–26. doi: 10.1111/cas.14149, PMID: 31361372 PMC6778640

[152] Baron JA Sandler RS Bresalier RS Quan H Riddell R Lanas A. A randomized trial of rofecoxib for the chemoprevention of colorectal adenomas. Gastroenterology. (2006) 131:1674–82. doi: 10.1053/j.gastro.2006.08.079, PMID: 17087947

[153] Brusselaers N Lagergren J. Maintenance use of non-steroidal anti-inflammatory drugs and risk of gastrointestinal cancer in a nationwide population-based cohort study in Sweden. BMJ Open. (2018) 8:e021869. doi: 10.1136/bmjopen-2018-021869, PMID: 29982219 PMC6042574

[154] Shekelle PG Newberry SJ FitzGerald JD Motala A O’Hanlon CE Tariq A. Management of Gout: a systematic review in support of an American College of Physicians Clinical Practice Guideline. Ann Intern Med. (2017) 166:37–51. doi: 10.7326/M16-0461, PMID: 27802478

[155] Shebl FM Hsing AW Park Y Hollenbeck AR Chu LW Meyer TE. Non-steroidal anti-inflammatory drugs use is associated with reduced risk of inflammation-associated cancers: NIH-AARP study. PLoS One. (2014) 9:e114633. doi: 10.1371/journal.pone.0114633, PMID: 25551641 PMC4281259

[156] Shimoji K Fujioka H. Pharmacology of analgesics. In: Chronic Pain Management in General and Hospital Practice. (2021). p. 55–86.

[157] Itani R Soubra L Karout S Rahme D Karout L Khojah HMJ. Primary dysmenorrhea: pathophysiology, diagnosis, and treatment updates. Korean J Fam Med. (2022) 43:101. doi: 10.4082/kjfm.21.0103, PMID: 35320895 PMC8943241

[158] Ouakrim DA Dashti SG Chau R Buchanan DD Clendenning M Rosty C. Aspirin, ibuprofen, and the risk of colorectal cancer in lynch syndrome. J Natl Cancer Inst. (2015) 107:djv170. doi: 10.1093/jnci/djv170, PMID: 26109217 PMC4651105

[159] Qaseem A Harris RP Forciea MA Denberg TD Barry MJ Boyd C. Management of acute and recurrent gout: a clinical practice guideline from the American College of Physicians. Ann Intern Med. (2017) 166:58–68. doi: 10.7326/M16-0570, PMID: 27802508

[160] Oyler DR Parli SE Bernard AC Chang PK Procter LD Harned ME. Nonopioid management of acute pain associated with trauma: focus on pharmacologic options. J Trauma Acute Care Surg. (2015) 79:475–83. doi: 10.1097/TA.0000000000000755, PMID: 26307883

[161] Testa U Castelli G Pelosi E. Genetic alterations of metastatic colorectal cancer. Biomedicine. (2020) 8:414. doi: 10.3390/BIOMEDICINES8100414 PMC7601984 33066148

[162] Chubak J Whitlock EP Williams SB Kamineni A Burda BU Buist DSM. Aspirin for the prevention of cancer incidence and mortality: systematic evidence reviews for the U.S. preventive services task force. Ann Intern Med. (2016) 164:814–25. doi: 10.7326/M15-2117, PMID: 27064482

[163] Boland PM Yurgelun MB Boland CR. Recent progress in lynch syndrome and other familial colorectal cancer syndromes. CA Cancer J Clin. (2018) 68:217–31. doi: 10.3322/caac.21448 29485237 PMC5980692

[164] Drew DA Cao Y Chan AT. Aspirin and colorectal cancer: the promise of precision chemoprevention. Nat Rev Cancer. (2016) 16:173–86. doi: 10.1038/nrc.2016.4, PMID: 26868177 PMC6741347

